# Arbuscular mycorrhizal hyphae facilitate rhizobia dispersal and nodulation in legumes

**DOI:** 10.1093/ismejo/wrae185

**Published:** 2024-09-26

**Authors:** Jiadong He, Lin Zhang, Judith Van Dingenen, Sandrien Desmet, Sofie Goormachtig, Maryline Calonne-Salmon, Stéphane Declerck

**Affiliations:** Laboratory of Mycology, Earth and Life Institute, Université catholique de Louvain-UCLouvain, Croix du Sud 2, L7.05.06B-1348, Louvain-la-Neuve, Belgium; College of Resources and Environmental Sciences, China Agricultural University, No. 2 Yuanmingyuan West Road, Haidian District, Beijing 100193, China; Department of Plant Biotechnology and Bioinformatics, Ghent University, Technologiepark 71, 9052 Ghent, Belgium; Center for Plant Systems Biology, VIB, Technologiepark 71, 9052 Ghent, Belgium; Department of Plant Biotechnology and Bioinformatics, Ghent University, Technologiepark 71, 9052 Ghent, Belgium; Center for Plant Systems Biology, VIB, Technologiepark 71, 9052 Ghent, Belgium; VIB Metabolomics Core, VIB, Technologiepark 71, 9052 Ghent, Belgium; Department of Plant Biotechnology and Bioinformatics, Ghent University, Technologiepark 71, 9052 Ghent, Belgium; Center for Plant Systems Biology, VIB, Technologiepark 71, 9052 Ghent, Belgium; Laboratory of Mycology, Earth and Life Institute, Université catholique de Louvain-UCLouvain, Croix du Sud 2, L7.05.06B-1348, Louvain-la-Neuve, Belgium; Laboratory of Mycology, Earth and Life Institute, Université catholique de Louvain-UCLouvain, Croix du Sud 2, L7.05.06B-1348, Louvain-la-Neuve, Belgium

**Keywords:** arbuscular mycorrhizal fungi, rhizobium, flavonoids, nodulation, gene expression, molecular docking

## Abstract

In soil ecosystems, rhizobia occupy the rhizosphere of legume roots to form nodules, a process triggered by microbial recognition of specific root-derived signals (i.e. flavonoids). However, soil conditions can limit bacterial motility, restricting signal perception to the area directly influenced by roots. Legumes, like most plants of agricultural interest, associate with arbuscular mycorrhizal fungi, whose hyphae develop extensively in the soil, potentially providing an effective dispersal network for rhizobia. We hypothesized that mycelial networks of arbuscular mycorrhizal fungi play a role in signal transmission and act as a highway, enabling rhizobia to migrate from distant soil to the roots of leguminous plants. Using *in vitro* and greenhouse microcosm systems, we demonstrated that *Rhizophagus irregularis* helps *Shinorhizobium meliloti* to migrate towards the legume *Medicago truncatula*, triggering nodulation, a mechanism absent without the arbuscular mycorrhizal fungus. Metabolomics analysis revealed eight flavonoids unique to the compartment containing extraradical hyphae of the arbuscular mycorrhizal fungus linked to *M. truncatula* roots, associated with *Sinorhizobium meliloti* growth and *nod* gene expression. Rhizobia plated on the extraradical hyphae connecting two plants (the legume *M. truncatula* and non-legume *Solanum tuberosum*) by a common mycelium network, showed preference for the legume, suggesting the chemoattraction by specific signals transported by the fungus connected to the legume. Simultaneously, *S. meliloti* stimulated the cytoplasmic/protoplasmic flow in the hyphae, likely increasing the release of nutrients and signals. Our results highlight the importance of extraradical hyphae (i.e. the mycorrhizal pathway) of arbuscular mycorrhizal fungi for the migration of rhizobia over long distances to the roots, leading to nodulation.

## Introduction

Leguminous crops have the ability to interact symbiotically with arbuscular mycorrhizal (AM) fungi and with nitrogen-fixing soil bacteria collectively known as rhizobia [1]. It has been shown that the contribution of AM fungi to N uptake was 27% in *Vicia faba* colonized by *Rhizophagus irregularis* in a pot experiment conducted on phosphorus-deficient soil [2], and even 52% in a two-compartment growth system of *Glycine max* L. colonized by *R. intraradices* [3]. Similarly the rhizobia association has been shown to mobilize high amounts of N, estimated to be ~65 to 335 kg of N·ha^−1^·year^−1^ for alfalfa, red clover, pea, soybean, cowpea, and vetch [4]. It is estimated that 70 million tons of N per year are contributed to soil ecosystems by the relationship between legumes and their rhizobial symbionts [5]. Along with rhizobia, AM fungi are thus considered to be the most crucial terrestrial symbioses that “help feed the world” [6].

Legume–rhizobia interactions are initiated when plant roots release flavonoids into the soil, the perception of which activates the production and secretion of rhizobial Nod factors. However, the perception of these signals is limited to a few millimeters in the rhizosphere [7] and several abiotic factors (e.g. soil organic matter, sorption by minerals) may attenuate flavonoid-based plant-microbe communication [8]. In addition, soil conditions can limit the mobility of rhizobia, subject to water mass flow and other vectors to cross air-filled gaps between soil aggregates [9, 10]. Until recently [7], it was unclear whether and how rhizobia migrate from long distance to the rhizosphere of legumes. These authors demonstrated that mycelial networks of the facultative biotrophic ascomycota *Phomopsis liquidambaris* help with the migration of rhizobia from the bulk soil to the rhizosphere of *Arachis hypogaea*, increasing nodulation in this legume. This study laid the foundation for the hypothesis that legumes might also recruit rhizobia via the extraradical mycelium (ERM) of AM fungi. Indeed, the hyphae of AM fungi extends several cm away from plant roots [11], represents 82 to 111 m·cm^−3^ in prairie and 52 to 81 m·cm^−3^ in ungrazed pasture [12] and mobilize between 4 and 20% of the total carbon synthesized by plants [13]. In addition, bacterial activities (e.g. the solubilization of organic P), are stimulated by the release of compounds or nutrients from the hyphae, which function as signals [14]. Recently, it has been demonstrated that bacteria could move along the hyphae to nutrient patches in the soil [15]. Hence, we hypothesized that rhizobia can also use the ERM of AM fungi as a highway to reach the roots of legumes from long distances, thus representing an indirect route for legumes to recruit root-nodulating bacteria.

In the present study, we performed a series of *in vitro* and greenhouse experiments to study the migration potential of *Sinorhizobium meliloti* along the ERM of AM fungi connected to the leguminous plant *Medicago truncatula* or the non-leguminous plants *Plantago lanceolata* and *Solanum tuberosum.* We first developed an *in vitro* cultivation system with *M. truncatula* to study whether AM fungal hyphae of *Rhizophagus irregularis* could transport rhizobia from a long distance from the root to the root surface to result in nodulation. Next, we conducted a comparative metabolomics study on the growth media containing AM fungal hyphae connected to *M. truncatula* or *S. tuberosum* in a compartmented system to reveal the presence and potential differences in nodule-inducing flavonoids content. Finally, we carried out an *in vitro* and a pot microcosm experiment with a leguminous plant connected by a common mycorrhizal network (CMN) of the AM fungus to non-leguminous plants to assess the preferred migration direction of rhizobia.

We show that AM fungal hyphae can serve as a route for *S. meliloti* migration, showing a preference for the legume over the non-legumes. This preference is probably due to the transport of specific signals (i.e. flavonoids) from the legume root to the bacteria via the hyphae, initiating the distant interaction between the legume and *S. meliloti*.

## Materials and methods

### Biological material

Experiments were performed with *Rhizophagus irregularis* MUCL41833 (ref. 13) and *Sinorhizobium meliloti 2011* pHC60-GFP [16] (Supplementary 1, Biological material). Seeds of *Medicago truncatula* L., cv. Jemalong A17 and *Plantago lanceolata* L. were provided by the South Australian Research and Development Institute (SARDI, Australia) and ECOSEM (Corroy-le-Grand, Belgium), respectively and nodal cuttings of *Solanum tuberosum* L. cv Bintje were supplied in the form of *in vitro* propagated plantlets by the Station de Haute Belgique in Libramont, Belgium (Supplementary 1, Biological material). The choice of *S. tuberosum* was based on the successful development of a stable and mature *in vitro* cultivation system for this species [17], which enabled our experiments to be carried out successfully.

### Optimization of MSR medium for growth and nodulation of *M. truncatula in vitro*

**Figure 1:**
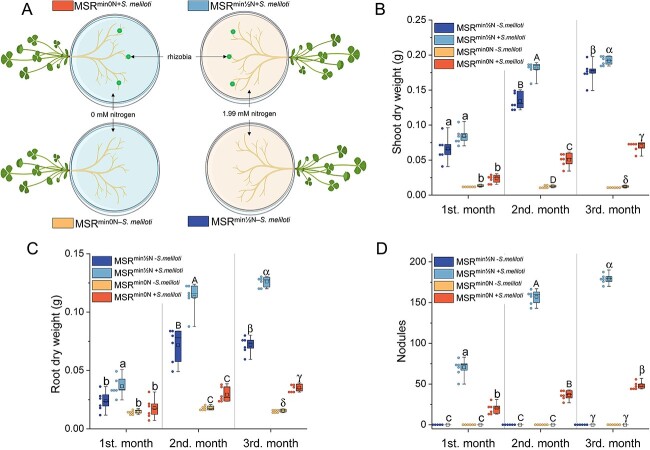
**Impact of *S. Meliloti* on the *in vitro* growth and nodulation of *M. truncatula* on the MSR** ^**min**^  **medium containing 0 mM or 1.99 mM nitrogen (N).** (**A**) Schematic representation of the *in vitro* petri plate system. Created in BioRender. (**B**) Shoots dry weight (**C**) roots dry weight, and (**D**) number of nodules, after 1, 2, and 3 months of growth of *M. Truncatula*. The box plot displays the maximum, upper quartile, median, lower quartile, minimum values. Hollow squares represent mean values, dots represent individual measurements. Data (n = 6) were analyzed by a one-way ANOVA followed by a Tukey post-hoc test (*P ≤* 0.05). Different lowercase letters, uppercase letters, and Greek letters, respectively, indicate significant differences between treatments within month 1, 2, or 3, separately.

Seven-days-old *M. truncatula* plants were grown in Petri plates (90 mm diam.) as detailed previously [17] (Fig. 1A; Fig. S1) and kept in a growth chamber at 22/18°C (day/night), 70% relative humidity, photoperiod of 16 h·day^−1^ and an average photosynthetic photon flux of 225 μmol·m^−2^·s^−1^. The nodulation was evaluated on two Modified Strullu-Romand medium [18] lacking sucrose and vitamins (MSR^min^) differing in N content: (1) MSR^min0N^ with 0 mM N and (2) MSR^min½N^ containing half the N concentration (1.99 mM) of the normal MSR^min^ medium (Supplementary 1, Composition of the Modified-Strullu-Romand medium). The roots were subsequently inoculated with 150 μl of a suspension of *S. meliloti* in PBS solution, at a concentration adjusted to 9 × 10^5^ CFU·ml^−1^. The controls received an equal volume of PBS solution without bacteria. Four treatments were thus considered with 18 replicates (i.e. plants) per treatment: MSR^min0N+*S.meliloti*^, MSR^min0N-*S.meliloti*^, MSR^min½N+*S.meliloti*^, and MSR^min½N-*S.meliloti*^. For each treatment, six replicates were randomly harvested after 1, 2, and 3 months to determine shoot and root dry weights (after 72 h at 70°C) and number of nodules.

### *In vitro* experimental design for analyzing flavonoids released by the ERM of *R. irregularis* in the HC

**Figure 2:**
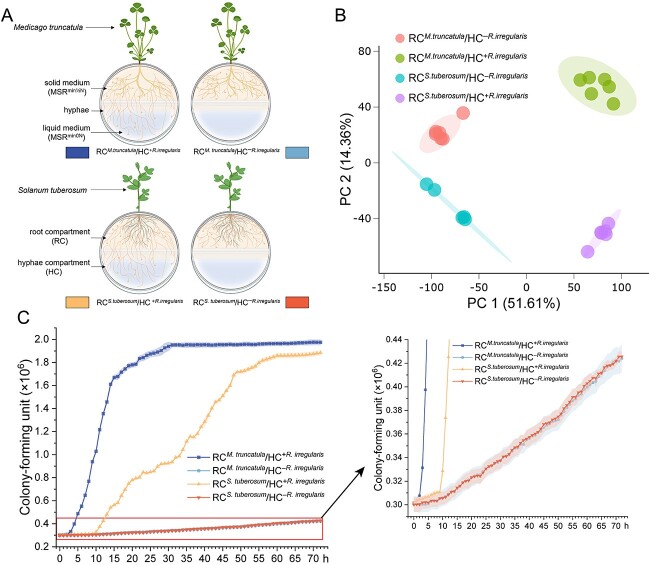
**Impact of ERM of *R. irregularis* on transport of flavonoids and on growth in *S. Meliloti*.** (**A**) Schema of the bi-compartmented petri plate system. The root compartment (RC) is separated from the hyphal compartment (HC) by a plastic barrier. *M. truncatula* Roots were placed in the RC with shoots extending outside through a 2 mm hole. RC contained 20 ml solidified MSR^min½N^ medium (1.99 mM N), and HC contained 4 ml solidified MSR^min0N^ medium (0 mM N) in a slope. Once profuse ERM growth was observed in the slope, 10 ml of liquid MSR^min0N^ medium lacking Ca(NO_3_)_2_·4H_2_O was added to the HC. Created in BioRender. (**B**) Principal component analysis (PCA) of compounds in the MSR^min0N^ medium collected from the HC. (**C**) Growth of *S. Meliloti* in liquid MSR^min0N^ medium from different treatments: RC*^M.truncatula^*/HC^+*R.irregularis*^, RC*^M.truncatula^*/HC^-*R.irregularis*^, RC*^S.tuberosum^*/HC^+*R.irregularis*^, And RC*^S.tuberosum^*/HC^-*R.irregularis*^. Data are means ± SD (n = 12, 72 h, OD = 595). Shaded areas represent error bars.

*Medicago truncatula* plants were pre-mycorrhized in the mycelium donor plant system [19] (Supplementary 1, MDP *in vitro* culture system; Fig. S2) and subsequently transferred to the RC of bi-compartmented Petri plates (90 mm diam.) (Fig. 2A; Fig. S3). At week 9, a profuse ERM growth was observed in the RC and on the slope in the hyphal compartment (HC) containing MSR^min½N^ medium. During the subsequent four weeks, 10 ml of MSR^min½^ medium cooled to 40°C was added weekly to the RC. Similarly, the HC was supplemented twice a week with liquid MSR^min0N^ medium to keep the medium volume at 10 ml. Four treatments were considered: *M. truncatula* or *S. tuberosum* grown in the RC in absence of AM fungus in the HC (RC*^M.truncatula^*/HC^–*R.irregularis*^ and RC*^S.tuberosum^*/HC^–*R.irregularis*^, respectively) and *M. truncatula* or *S. tuberosum* grown in the RC in presence of the AM fungus in the HC (RC*^M.truncatula^*/HC^+*R.irregularis*^ and RC*^S.tuberosum^*/HC^+*R.irregularis*^, respectively). At week 13, a high number of hyphae was observed in the HC. At that time, the liquid medium in the HC was removed and 10 ml of fresh liquid MSR^min0N^ was added for an extra week. At week 14, all the liquid MSR^min0N^ medium was collected with a VWR Powerpette Plus and transferred to a 15 ml centrifuge tube, quickly frozen in liquid N, and stored at −80°C for flavonoids analysis (Supplementary 1, Flavonoids analysis in the HC colonized by *R. irregularis*).

### *In vitro* experimental design for analyzing growth and gene expression of *S. Meliloti* in presence of ERM exudates

Another set of 24 Petri plates initiated simultaneously under identical conditions to above. At week 14, two ml liquid MSR^min0N^ was collected from the HC of each treatment to study its effects on the growth of *S. meliloti*. In parallel, two ml of *S. meliloti* at a concentration of 5.4 × 10^6^ CFU·ml^−1^ was added to the HC in each treatment. Samples were taken at 0, 2, 4, 6, 12, and 24 h, frozen in liquid N, and stored at −80°C for RNA extraction and gene expression analysis. Growth measurements of *S. meliloti* were performed using a Multiskan FC Microplate Photometer (Thermo Fisher Scientific Inc., Waltham, MA) at 30°C for 72 h, recording absorbance at OD_595_ every hour (Supplementary 1, *S. meliloti* growth and gene expression analyze).

### *In vitro* experimental design for the analysis of *S. Meliloti* migration along AM fungal hyphae and cytoplasmic flow velocity within hyphae

**Figure 3:**
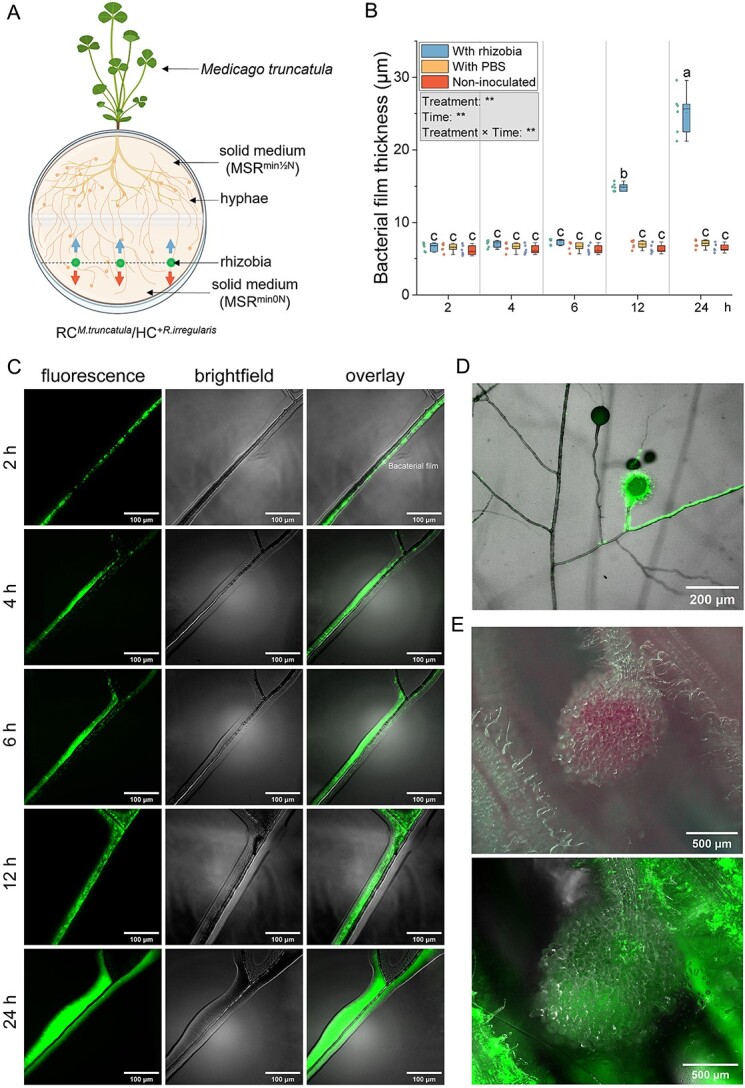
**Interactions between *R. irregularis* hyphae and *S. meliloti*.** (**A**) Schema of the bi-compartmented petri plate system used to study the impact of ERM on *S. meliloti* migration. The system includes a root compartment (RC) and a hyphal compartment (HC) separated by a plastic barrier. *M. truncatula* Roots were placed in the RC, with shoots extending through a 2 mm hole. RC contained 20 ml MSR^min½N^ medium (1.99 mM N), and HC contained 20 ml MSR^min0N^ medium (0 mM N). Dots in the HC indicate *S. Meliloti* inoculation sites; upward and downward arrows show the directions of bacterial growth along hyphae towards and away from the RC. Created in BioRender. (**B**) Bacterial film thickness (μm) of *R. irregularis* inoculated with *S. Meliloti* or PBS or non-inoculated, measured at 2, 4, 6, 12, and 24 h. the box plot displays the maximum, upper quartile, median, lower quartile, minimum values. Hollow squares represent mean values, dots represent individual measurements. Data (n = 6) were analyzed by a one-way ANOVA followed by a Tukey post-hoc test (*P ≤* 0.05). Different lowercase letters above the box plots indicate significant differences among each treatment. In the gray box, the results of the two-way ANOVA on “treatment” and “time” are presented, where ** represents *P ≤* 0.01. (**C**) *S. meliloti* migration along *R. irregularis* hyphae in the HC of petri plates with *M. truncatula* in the RC, shown under fluorescence, brightfield, and overlay views at 2, 4, 6, 12, and 24 h. (**D**) Microscopic picture of *S. meliloti* colonizing the surface of spores. (E) Microscopic picture of the nodule formed on the root of *M. truncatula*. The upper picture is the mature nodule under bright-field microscopy, and the lower picture is the nodule under fluorescent microscopy.

The above experimental design was also used with the HC consisting of solid MSR^min0N^ medium (Fig. 3A). At week 13, ~80% of the HC surface was covered with an extensive ERM. Three hyphae in each HC were inoculated with 1 μl of *S. meliloti* at 9 × 10^5^ CFU·ml^−1^, and speed of colonization quantified by measuring the distance traveled by *S. meliloti* along the hyphae from the point of inoculation divided by the time post-inoculation (2, 4, 6, 12, and 24 h). Six replicates were used per treatment. Another set of 18 Petri plates were used to evaluate the cytoplasmic flow velocity within hyphae. Three hyphae per plate were inoculated as above. Two control treatments were included: hyphae inoculated with PBS and non-inoculated hyphae. Six replicates per treatment were used. Images and videos were acquired using an Echo Revolve RVL2-K microscope (San Diego, CA, USA) with at least a 1/4 overlap between adjacent images. Images were stitched using Fiji’s Stitching plugin [20]. Migration of *S. meliloti* along hyphae and the thickness of the bacterial film around hyphae were measured using Fiji’s Measure tool [20]. Flow velocity was calculated by measuring the distance traveled by particles within hyphae in video frames. Six measurements per video from six replicates were averaged (Supplementary 1, *S. meliloti* migration along AM fungal hyphae and cytoplasmic flow velocity within hyphae).

### *In vitro* and greenhouse experimental designs with legumes linked to non-legumes by a CMN for mycelia-based migration assay of *S. meliloti*

**Figure 4:**
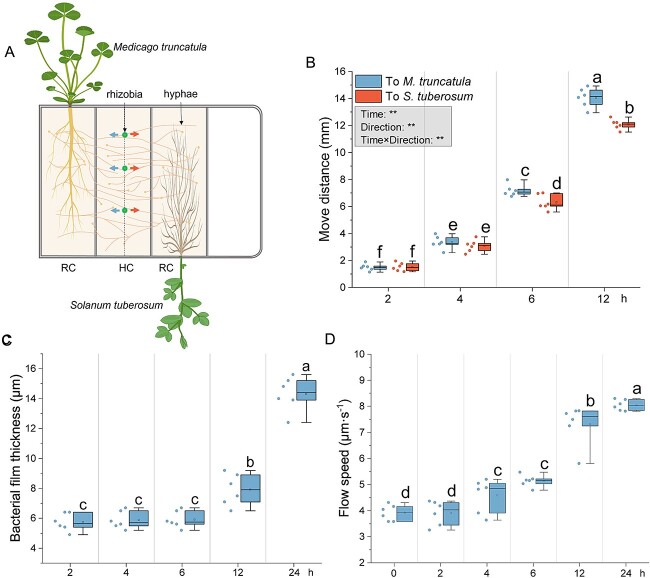
**Migration of *S. meliloti* on the surface of hyphae connecting a leguminous plant (*M. truncatula*) to a non-leguminous plant (*S. tuberosum*).** (**A**) Schema of the *in vitro* tri-compartmented system. This system uses three compartments of a quadri petri plate: Two root compartments (RCs) containing *M. truncatula* and *S. tuberosum* and a hyphal compartment (HC) where hyphae connect the two plants. After 13 weeks, hyphae from both RCs crossed into the HC, forming numerous anastomoses. *S. meliloti* Was then added to the surface of hyphae in the middle of the HC, and its migration was measured in both directions. Dots in the HC indicate *S. meliloti* inoculation points. Left-pointing arrows show bacterial growth towards *M. truncatula*; right-pointing arrows show growth towards *S. tuberosum*. Created in BioRender. (**B**) Migration distance (mm) of *S. meliloti* along hyphae towards *M. truncatula* and *S. tuberosum*. (**C**) Effects of *S. meliloti* on bacterial film thickness (μm) at 2, 4, 6, and 24 h. (**D**) Cytoplasmic/protoplasmic flow speed (μm·s^−1^) in *R. irregularis* hyphae connecting *M. truncatula* and *S. tuberosum*, measured at 0, 2, 4, 6, and 24 h post-inoculation. The box plot displays the maximum, upper quartile, median, lower quartile, minimum values. Hollow squares represent mean values, dots represent individual measurements. Data (n = 6) were analyzed by a one-way ANOVA followed by a Tukey post-hoc test (*P ≤* 0.05). Different lowercase letters above the box plots indicate significant differences among each treatment. In the gray box, the results of the two-way ANOVA on “treatment” and “time” are presented, where ** represents *P ≤* 0.01.

***In vitro experimental design—*** A CMN of *R. irregularis* was established between *M. truncatula* and *S. tuberosum* in a modified quadri-compartmented Petri plate, using only three compartments: two side RCs containing *M. truncatula* in one RC and *S. tuberosum* in the other RC, associated to *R. irregularis*, both separated from a central HC by plastic barriers (Fig. 4A; Fig. S4). The RCs contained MSR^min½N^ medium, and HC contained MSR^min0N^ medium. At week 13, the ERM in the two RCs crossed the plastic barriers into the HC, forming a CMN. Three hyphae per system were inoculated with 1 μl of *S. meliloti* at 9 × 10^5^ CFU·ml^−1^. Measurements included the distance moved by *S. meliloti* towards *M. truncatula* or *S. tuberosum*, cytoplasmic flow velocity, and bacterial film thickness around hyphae were evaluated on six replicates (Supplementary 1, *In vitro* experimental design with legumes linked to non-legumes).

**Figure 5:**
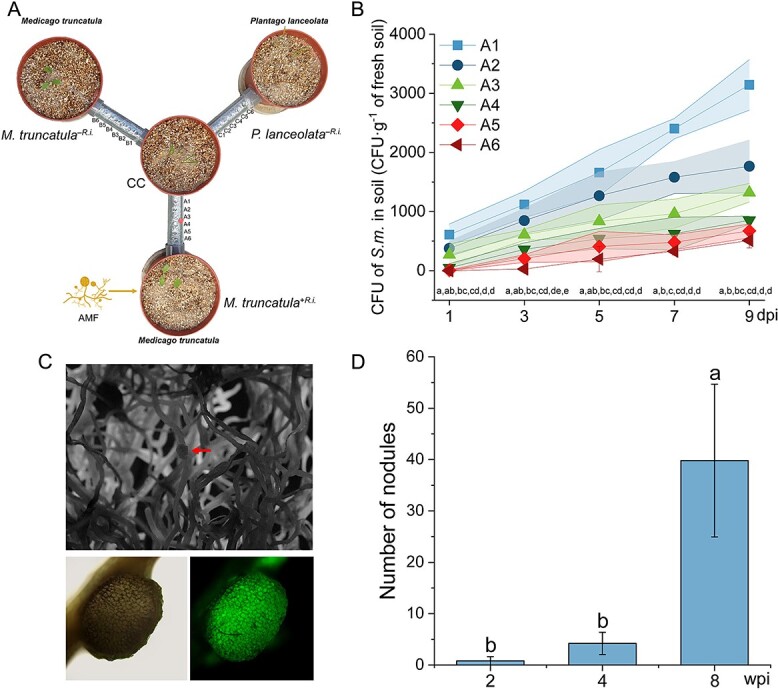
**Impact of** ERM **of the AM fungus on the migration of *S. meliloti* in a quadri-compartmented pot device.** (**A**) Schema of the quadri-compartmented pot device. *P. lanceolata* was planted in the CC, whereas *M. truncatula* (with or without AM fungus, *M. truncatula*^+*R.i.*^ and *M. truncatula*^–*R.i.*^) and *P. lanceolata* (without AM fungus, *P. lanceolata*^–*R.i.*^) were planted in three satellite compartments. These were connected to the CC via PVC pipes (1.7 cm diameter, 10 cm long) with six evenly spaced 6 mm holes for substrate sampling (Fig. S8). The *M. truncatula*^+*R.i.*^ compartment was connected to the CC with pipes covered by a 41 μm mesh to allow only hyphae passage. The other compartments were connected via pipes covered with 5 μm mesh to block both hyphae and roots. *P. lanceolata* in the CC was used to confirm hyphal growth into the CC; after confirmation, the plants were removed. After one month of ERM regrowth, *S. meliloti* was inoculated in the CC, and substrate samples were collected over time to assess rhizobia motility. (**B**) Concentration of *S. meliloti* (CFU·g^−1^ fresh substrate) in the different holes (A1 to A6) of the pipes connecting the CC to the *M. truncatula*^+*R.i.*^ pots at 1, 3, 5, 7, and 9 dpi. Data are means ± SD (n = 5). Shaded areas represent error bars. (**C**) Microscopic pictures of nascent nodules developing on *M. truncatula*^+*R.i.*^ roots two weeks after inoculation with *S. meliloti* in the CC. (**D**) Average number of nodules on *M. truncatula*^+*R.i.*^ roots at 2, 4, and 8 weeks post-inoculation. Data (means ± SD, n = 5) were analyzed by one-way ANOVA followed by the Tukey post-hoc test (*P ≤* 0.05). Different lowercase letters above the bars indicate significant differences among each treatment. No nodules were observed in *M. truncatula*^-*R.i.*^ and *P. lanceolata*^-*R.i.*^ treatments throughout the experiment (data not shown in the figure).

***Greenhouse experimental design—*** A quadri-compartmented pot system was created with a central compartment (CC) connected to three satellite compartments via perforated pipes. *P. lanceolata* was planted in the CC to monitor the AM fungi, and the satellite compartments contained *M. truncatula* with or without *R. irregularis* and *P. lanceolata* without *R. irregularis* (Fig. 5A; Fig. S7). The substrate was composed of a mixture of sand and vermiculite in a 2:1 volume ratio with two times autoclaving (0.11 MPa, 121°C, 15 min per time). The *M. truncatula*^+*R.i.*^ compartment received 5 g of *R. irregularis* inoculum, and pipes were sealed with 41 μm mesh to allow only hyphae proliferation. The other pipes were sealed with 5 μm mesh to prevent hyphae and roots from crossing. After root colonization was confirmed, plants in the CC were removed. *S. meliloti* (5 ml of 9 × 10^6^ CFU·ml^−1^) was inoculated in the CC, and substrate samples were collected from the pipes over 9 days to evaluate *S. meliloti* migration. Samples were processed and CFUs counted. Fresh roots were assessed for AM fungal colonization, and hyphal length in the pipes was measured. Nodules were evaluated in the satellite compartments for maturity and quantity (Supplementary 1 Greenhouse experimental design with legumes linked to non-legumes).

### Data analysis

Prior to statistical analysis, the homogeneity of variance and normality of distribution were assessed using the Levene and Shapiro–Wilk tests, respectively. To attain normality and homoscedasticity of data, all the variables were transformed by taking the base 10 logarithm. Analysis of variance was conducted in SAS (8.1) to evaluate the statistical significance of the experimental data. Significance between treatments was determined at 0.05 using the Tukey post-hoc test.

## Results

### *S. meliloti* promotes the growth of *M. truncatula* on the AM fungi-compatible MSR medium containing reduced or no N

We first tested whether the MSR^min^ medium developed for growing AM fungi on root organs [18] or whole plants [21] was adequate for the growth and nodulation of *M. truncatula* in the presence of *S. meliloti.* We compared the growth and nodulation of the plants associated or not to the bacterium on the MSR^min0N^ and MSR^min½N^ media (Fig. 1A; Fig. S1). In the MSR^min0N-*S.meliloti*^ treatment, the growth of *M. truncatula* was strongly limited (Fig. 1B, C), and no nodule was observed (Fig. 1D). Conversely, in the presence of *S. meliloti*, shoot and root dry weight increased significantly in the MSR^min½N+*S.meliloti*^ treatment, with increases of 253.6% and 104.9% in month 1, 256.0% and 285.2% in month 2, and 174.2% and 259.1% in month 3, respectively, compared to the MSR^min0N+*S.meliloti*^ treatment (Fig. 1B, C). Whatever the time of observation, the number of nodules was three to four times higher in the MSR^min½N+*S.meliloti*^ treatment than in the MSR^min0N+*S.meliloti*^ treatment (Fig. 1D). Overall, it appeared that the MSR^min½N^ medium is suitable for the growth and nodulation of *M. truncatula* in presence of *S. meliloti* and was therefore selected for all following *in vitro* experiments.

### ERM transports flavonoids from roots to medium

We used a bi-compartmented system separating AM fungal-colonized plants (RC) from ERM of *R. irregularis* (HC) (Fig. 2A) to assess flavonoid transport by hyphae. LC–MS/MS analysis of the HC revealed 20 081 m/z features across four treatments. Principal component analysis (PCA) (Fig. 2B) showed distinct clustering, i.e. the samples from both plant species, both in the presence or absence of the AM fungus, are clustering separately, indicating the crucial influence of AM fungal hyphae on the transport of plant compounds such as flavonoids.

We retained features with a minimum intensity of 1000 counts in at least one sample group, resulting in 13 513 features. ANOVA analysis (*P* ≤ 0.05) identified 11 662 significant features. MS-FINDER structural annotation of these features recorded MS/MS spectra for 2056 features, identifying 441 potential metabolites, with 23 annotated as flavonoids (Supplemental dataset 1). Eight flavonoids were found exclusively in RC*^M.truncatula^*/HC^+*R.irregularis*^ treatment, suggesting transport from *M. truncatula* roots via AM fungal hyphae. One of these was a luteolin derivative, known to attract *S. meliloti* and induce nodulation genes [22]. Three compounds were present in both *RC^M.truncatula^*/HC^+*R.irregularis*^ and RC*^S.tuberosum^*/HC^+*R.irregularis*^ treatments, and two features were unique to RC*^S.tuberosum^*/HC^+*R.irregularis*^ treatment.

### ERM exudates impact the growth and gene expression in *S. meliloti*

We investigated the influence of exudates from ERM in the HC connected to *M. truncatula* or *S. tuberosum* in the RC on *S. meliloti* growth and nodulation genes expression. The growth of *S. meliloti* was analyzed over a 72-h period (Fig. 2C). In treatments without hyphae in the HC (control), slow bacterial growth was observed with no significant differences between RC*^M.truncatula^*/HC^–*R.irregularis*^ and RC*^S.tuberosum^*/HC^–*R.irregularis*^ treatments. Conversely, with hyphae in the HC (with ERM exudates), *S. meliloti* growth was significantly higher. The growth pattern was sigmoidal, with a minimal increase during the first 4 and 10 h for RC*^M.truncatula^*/HC^+*R.irregularis*^ and RC*^S.tuberosum^*/HC^+*R.irregularis*^ treatment, respectively (Fig. 2C), followed by exponential growth, plateauing at 30 and 60 h, respectively. From the 3^rd^ h onward, CFUs in RC*^M.truncatula^*/HC^+*R.irregularis*^ exudates were significantly higher than with RC*^S.tuberosum^*/HC^+*R.irregularis*^ exudates. These results indicate that ERM exudates stimulate bacterial growth, especially when connected to the legume.

**Figure 6:**
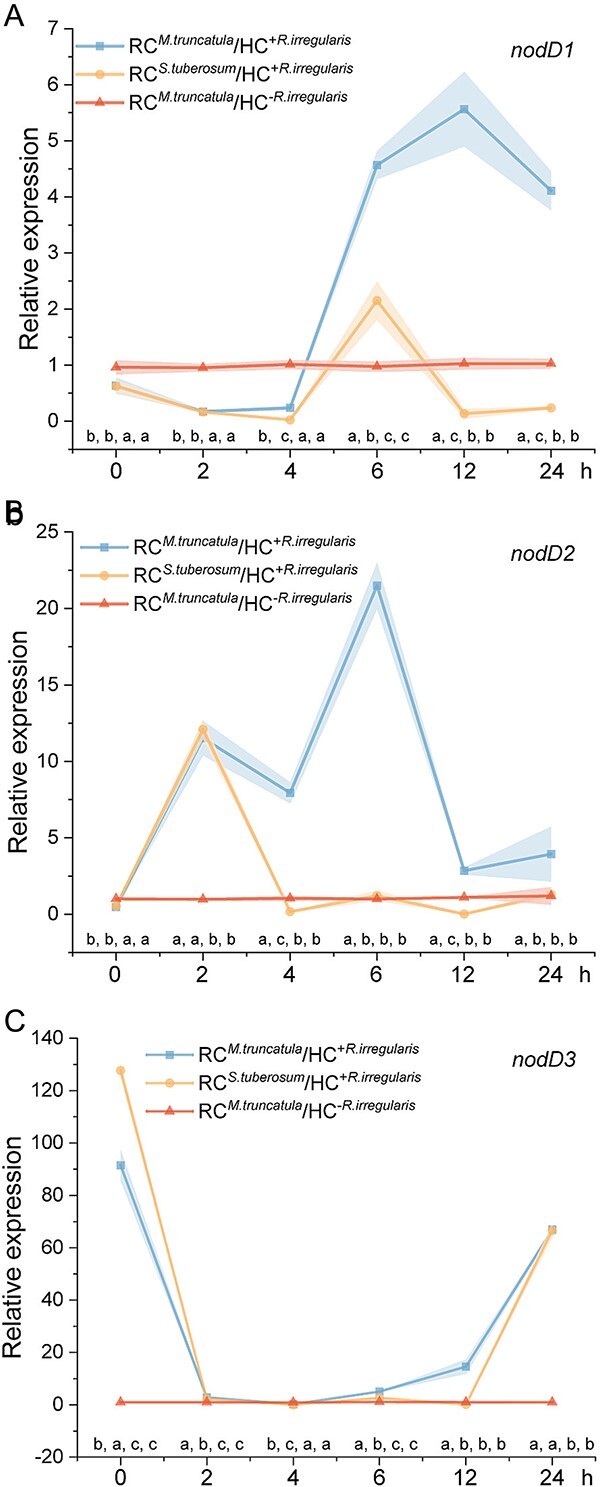
**Impact of ERM exudates on the *nod* gene expression of *S. meliloti*.** Relative expression of *nodD1* (**A**), *nodD2* (**B**), and *nodD3* (**C**) in *S. meliloti* at 0, 2, 4, 6, 12, and 24 h after inoculation in the hyphal compartment (HC) of four treatments: RC*^M.truncatula^*/HC^+*R.irregularis*^, RC*^M.truncatula^*/HC^–*R.irregularis*^, RC*^S.tuberosum^*/HC^+*R.irregularis*^, And RC*^S.tuberosum^*/HC^–*R.irregularis*^. Expression values were normalized to the RC*^S.tuberosum^*/HC^–*R.irregularis*^ treatment (not shown). Shaded areas represent error bars. Data (n = 6) are means ± SD and were analyzed by one-way ANOVA with Tukey’s post-hoc test (*P ≤* 0.05). Different lowercase letters above each time point on the X-axis indicate significant differences between treatments at that time point, with the letters in sequence representing RC*^M.truncatula^*/HC^+*R.irregularis*^, RC*^M.truncatula^*/HC^–*R.irregularis*^, RC*^S.tuberosum^*/HC^+*R.irregularis*^, And RC*^S.tuberosum^*/HC^–*R.irregularis*^.

We further investigated whether the MSR^min0N^ medium in the HC containing hyphae connected to *M. truncatula* or *S. tuberosum* in the RC could trigger Nod factor expression in *S. meliloti*. The expression of eight *nod* genes (*nodD1*, *nodD2*, *nodD3, nodA*, *nodB*, *nodC*, *nodI*, and *nodJ*) were analyzed at 0, 2, 4, 6, 12, and 24 h after adding the bacterium to the medium (Fig. 6; Fig. S5). No significant differences in gene expression were observed between the two control treatments at any time point.

Focusing on the *nodD* genes, which code for the transcription factor NodD, we found that *nodD1* expression was significantly higher in the RC*^M.truncatula^*/HC^+*R.irregularis*^ treatment compared to both controls and the RC*^S.tuberosum^*/HC^+*R.irregularis*^ treatment at 6, 12, and 24 h. In the RC*^S.tuberosum^*/HC^+*R.irregularis*^ treatment, *nodD1* expression was significantly higher at 6 h but lower at other times compared to both controls (Fig. 6A). For *nodD2*, expression was significantly higher in the RC*^M.truncatula^*/HC^+*R.irregularis*^ treatment at 2, 4, 6, 12, and 24 h compared to both controls, and except at 0 and 2 h, it was also higher than in the RC*^S.tuberosum^*/HC^+*R.irregularis*^ treatment. In the RC*^S.tuberosum^*/HC^+*R.irregularis*^ treatment, *nodD2* expression was only significantly higher than both controls at 2 h (Fig. 6B). The *nodD3* expression was significantly higher in the RC*^M.truncatula^*/HC^+*R.irregularis*^ treatment at 0, 2, 6, 12, and 24 h, and in the RC*^S.tuberosum^*/HC^+*R.irregularis*^ treatment at 0, 2, 6, and 24 h compared to both controls. Additionally, the RC*^S.tuberosum^*/HC^+*R.irregularis*^ treatment showed higher *nodD3* expression compared to the RC*^M.truncatula^*/HC^+*R.irregularis*^ treatment at 0 h, with the reverse observed at later times (Fig. 6C). The structural *nod* genes were further analyzed, and results reported in Supplementary 1 (Expression of structural *nod* genes).

**Figure 7:**
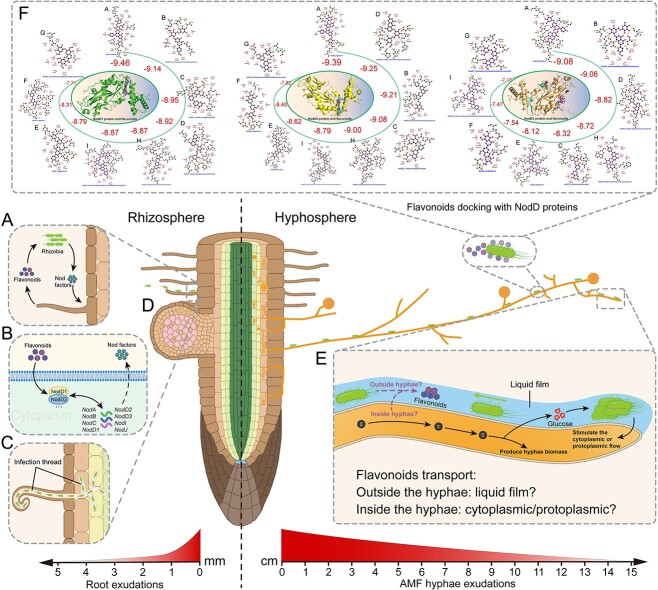
**Schematic representation of the two pathways for AM fungi-colonized legumes to recruit rhizobia.** In the direct pathway (**left side**), root exudates mediate the short-distance recruitment (a few millimeters) of rhizobia via the release of flavonoids (**A**), the perception of which activates the production and secretion of rhizobial nod factors (**B**), which subsequently stimulate the root hairs to curl around the rhizobia, penetrating the hairs via an infection thread (**C**), ultimately stimulating the formation of the nodule (**D**). In the indirect pathway (**right side**), the ERM of the AM fungus connected to the legume release sugars (i.e. glucose), stimulating rhizobial growth, and flavonoids, acting as signal molecules allowing the bacteria to sense the presence of a compatible host at far distances in the soil (> 10 cm) (**E**). In return, the rhizobia stimulate the cytoplasmic/protoplasmic flow increasing the release of exudates (i.e. nutrients and signals). A gradient of exudates/signals is established from roots to hyphal tips, attributable to the combination of (i) a dilution effect of exudates from base to tip of hyphae, (ii) a decrease in number and density of hyphae, (iii) a combination of root hairs and potentially hyphae release of exudates in the rhizosphere. This gradient plays a role as chemoattractant allowing the bacteria to move in the direction of the host plant. At this stage it is unknown whether the transport of flavonoids by hyphae is via a liquid film at the hyphae surface or via cytoplasmic/protoplasmic streaming within the hyphae. In silico binding studies of NodD1 (left), NodD2 (middle), and NodD3 (right) proteins of *S. meliloti* and 10 flavonoids (**F**). Here NodD–flavonoid complexes were arranged based on their binding affinities (the more negative the values, (kcal·mol^−1^), the stronger the interactions). Each NodD–flavonoid complex form hydrogen (dotted line between amino acid and flavonoid) and hydrophobic (half circles) bonds during NodD–flavonoid interaction. The central picture (inside the oval circle) predicts the binding sites of 9 flavonoids in NodD protein of *S. meliloti*. Individual figures are shown below. A: Sagittain B1 and sagittain B2; B: Isosakuranetin-hexosyl-deoxyhexose; C: Luteolin-acetylhexuronide; D: Taxifolin-deoxyhexosyl-oxanepentol; E: Haploside C; F: Sudachiin a; G: Tetrahydroxy-tetramethoxyflavone-hexoside; H: Trihydroxy-flavanone-hexosyl-pentose; I: Trimethoxy-hydroxy-methyl-flavanone-dihexosyl-deoxyhexose. Sagittain B1 and Sagittain B2 were merged into a single flavonoid as their comparison results in the PubChem (https://pubchem.ncbi.nlm.nih.gov/) were identical. The H and I are exclusively found in the RC*^S.tuberosum^*/HC^+*R.irregularis*^ treatment. Created in BioRender.

We then conducted a *in silico* binding study between eight flavonoids from RC*^M.truncatula^*/HC^+*R.irregularis*^ treatment and two from RC*^S.tuberosum^*/HC^+*R.irregularis*^ treatment with NodD1, NodD2, and NodD3 (Fig. 7F; Supplementary 2). Among all the 10 flavonoids, sagittain B exhibited the most stable binding, with binding energies of −9.46, −9.39, and −9.08 Kcal·mol^−1^ for NodD1, NodD2, and NodD3, respectively. These results suggest that sagittain B, which is particularly present in RC*^M.truncatula^*/HC^+*R.irregularis*^, has a particularly strong binding affinity for NodD factors, suggesting a potentially dominant role in *S. meliloti* chemotaxis.

### *S. meliloti* efficiently colonizes the surface of *R. irregularis* hyphae and spreads rapidly along the ERM network to form nodules in *M. truncatula*

To investigate the interactions between *S. meliloti* and the ERM of *R. irregularis*, we examined the movement of *S. meliloti* along the hyphae connected to *M. truncatula* (Fig. 3A; Fig. S3). Using time-lapse microscopy imaging, we observed that *S. meliloti* inoculated onto hyphae moved as motile cells and formed thick (6.1 to 22.9 μm) multicellular colonies, resembling a biofilm (Fig. 3B, C). The cells also colonized spore surfaces, forming multilayers (Fig. 3D). After 14 d, the bacteria inoculated on the hyphae in the HC had migrated to the RC, forming functional nodules (Fig. 3E).

**Figure 8:**
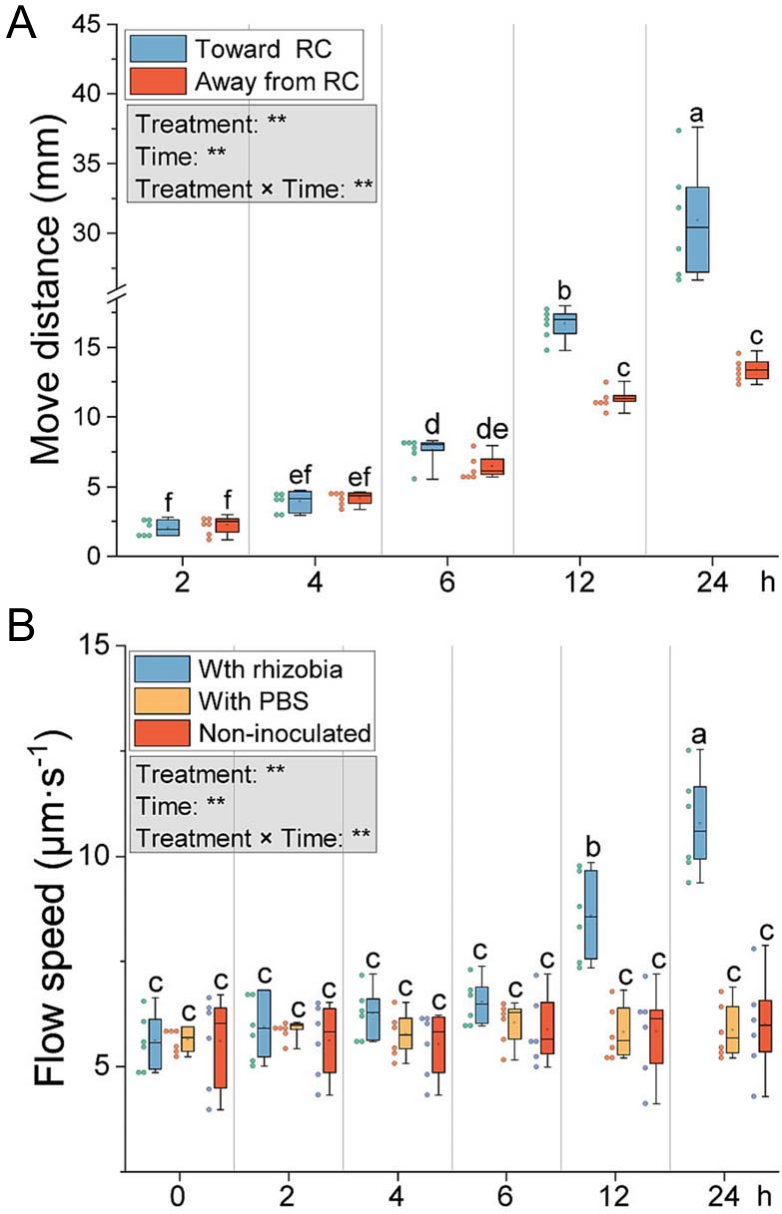
**Microbial migration and cytoplasmic flow in mycorrhizal hyphae.** (**A**) Migration of *S. meliloti* along *R. irregularis* hyphae in the HC connected to *M. truncatula* in the RC. Migration was measured towards the root (toward RC) and away from the root (away from RC) at 2, 4, 6, 12, and 24 h after bacterial plating on the hyphae surface. (**B**) Cytoplasmic/protoplasmic flow within *R. irregularis* hyphae inoculated with *S. meliloti*, PBS or non-inoculated, measured at 2, 4, 6, 12, and 24 h. The box plot displays the maximum, upper quartile, median, lower quartile, minimum values. Hollow squares represent mean values, dots represent individual measurements. Data (n = 6) were analyzed by a one-way ANOVA followed by a Tukey post-hoc test (*P ≤* 0.05). Different lowercase letters above the box plots indicate significant differences among each treatment. In the gray box, the results of the two-way ANOVA on “treatment” and “time” are presented, where ** represents *P ≤* 0.01.

We analyzed *S. meliloti* migration along hyphae towards plant roots in the RC. Starting 12 h post-inoculation, *S. meliloti* migrated 47.1% farther toward RC than away from RC (Fig. 8A; Fig. S6; Video S1). The velocity of cytoplasmic flow in the hyphae significantly increased at 12 and 24 h after inoculation, with no significant changes observed in the PBS and non-inoculated treatments (Fig. 8B; Video S2).

### Hyphae connecting *M. truncatula* to *S. tuberosum* favor the migration of *S. meliloti* towards the legume

To further investigate the role of plant-connected AM fungal hyphae in recruiting rhizobia, a tri-compartment setup (Fig. 4A; Fig. S4) was used. Microscopic observations indicated that rhizobia preferentially migrate towards *M. truncatula* at 6 and 12 h (Fig. 4B). Bacterial multilayer thickness increased significantly at 12 h and was 1.8 times thicker at 24 h, indicating rapid proliferation (Fig. 4C). Cytoplasmic flow velocity in hyphae increased from 4 h post-inoculation, suggesting high nutrient demand by proliferating rhizobia (Fig. 4D).

In the quadri-compartment pot device (Fig. 5A; Fig. S7), the CMN was well established, with 82.5 ± 4.4% total colonization and 30.3 ± 4.3% arbuscule colonization in *P. lanceolata* within the CC (Table S1). This provided a solid foundation for studying *S. meliloti*’s ability to migrate along AM fungal hyphae. Bacterial migration along hyphae significantly increased, with 196 CFU·g^−1^ in hole A6 by Day 5 (Fig. 5B). Without ERM, CFUs were almost 0 CFU·g^−1^ in hole A6 (Fig. S8). Fourteen days post-inoculation, the first nodule appeared (Fig. 5C, D). After 4 weeks, *M. truncatula*^+*R.i*.^ had 4 ± 2 nodules per pot (10.5% mature), and after 8 weeks, 40 ± 15 nodules (63.1% mature) (Fig. 5D). No nodules were observed in treatments without ERM, indicating that AM fungal hyphae are a primary pathway for the migration of rhizobia over long distances to the roots, leading to nodulation.

## Discussion

The extraradical hyphae of AM fungi extend from plant roots to the soil, where they interact with microbial communities in the hyphosphere (the narrow zone of the soil influenced by the hyphae). This interface shapes microbial communities [23] and aids plants in acquiring nutrients such as P [14] and N [24]. Studies indicate an increase in legume nodulation when colonized by AM fungi [25, 26], suggesting these microorganisms enrich the rhizosphere with competent rhizobia. However, the mechanisms by which AM fungal hyphae facilitate the movement of N-fixing rhizobia along their surface towards legume roots remain largely unknown. Here we have developed *in vitro* plate systems to test the migration of *S. meliloti* to leguminous and non-leguminous plants connected to AM fungi, and a greenhouse-based quadri-compartment pot device to test this migration in the presence or absence of AM fungal ERM connections. We demonstrated that, in addition to the short-distance direct pathway via root epidermal cells and root hairs releasing flavonoids [7, 27], there is an indirect “mycorrhizal” pathway. In this pathway, rhizobia is able to move over long distances along the hyphae surface to the roots, leading to nodulation (Fig. 5).

Similar to root exudates in mediating plant–microorganism interactions, fungal hyphae release C-rich compounds like sugars and organic acids into their environment [24, 25], feeding the microorganisms developing at their surface. Some exudates also act as signaling molecules, such as fructose from *R. irregularis*, which triggers phytate mineralization by phosphate-solubilizing bacteria [14]. In legume-rhizobia interactions, flavonoids released by plant roots are essential signaling molecules that activate *nod* gene transcription and Nod factor synthesis in rhizobia, leading to nodulation [28–31]. Our metabolomics approach compared flavonoid compounds transported by hyphae connected to *M. truncatula* and *S. tuberosum*. Flavonoids were detected in the hyphae-containing HC of both plants. However, eight unique flavonoids were found in the HC connected to *M. truncatula*, including three flavonol glycosides, two flavanone glycosides, two flavone glycosides, and one flavone glucuronide. Quercetin-3-O-galactoside and luteolin-7-O-glucoside (flavone glycosides) are known to promote *S. meliloti* growth [32], which may explain the increased growth observed in the RC*^M.truncatula^*/HC*^+R.irregularis^* treatment (Fig. 2C). In contrast, only two kinds of flavanone glycosides were detected exclusively in the HC connected to *S. meliloti*. It remains unclear whether these compounds were transported inside the hyphae or along their surface.

Luteolin, which induces nodulation gene expression in *S. meliloti* [22, 33], was detected as luteolin-acetylhexuronide in our study. Our metabolomic analysis showed that flavonol glycosides, which stimulate rhizobia, were exclusive to the RC*^M.truncatula^*/HC*^+R.irregularis^* treatment. *NodD1*, *nodD2*, and *syrM2* regulate the flavonoid-induced symbiotic gene expression and nodule growth [34]. External addition of isoflavones enhances nod-lacZ fusion expression in *Bradyrhizobium japonicum* [35]. Therefore, we hypothesize that flavonol glycosides may play an important role in regulating *nod* gene expression in *S. meliloti*. Further investigation, such as gene knockout studies or reporter gene assays, is needed to elucidate their precise mechanisms and effects.

NodD, beyond activating *nod* genes, indirectly influences chemotaxis by regulating genes involved in exopolysaccharide production, flagellar function, and chemotaxis receptors like McpV, facilitating rhizobial movement toward host plants [36–38]. NodD1 and NodD2 are the main flavonoid-sensing regulatory factors in *S. meliloti* [38–40]. These highly homologous proteins directly respond to flavonoids secreted by host plant roots, activating the expression of *nod* genes, a crucial step in root nodule formation [40, 41]. In contrast, NodD3 functions through the transcriptional regulator SyrM, exerting its role under specific conditions and activating *nod* gene expression even in the absence of flavonoids [38, 42]. This observation highlights the greater importance of NodD1 and NodD2 in flavonoid sensing, as they directly participate in the response to host plant signals. To validate the function of NodD binding to flavonoids, we performed *in silico* binding studies (Supplementary 2). More negative binding affinity indicates stronger interaction [43]. Due to the high homology between NodD1 and NodD2 proteins, our molecular docking analysis revealed that all flavonoids bind to the same region of these proteins (Supplementary 2). Among the 10 flavonoids detected in the RC*^M.truncatula^*/HC^+*R.irregularis*^ and RC*^S.tuberosum^*/HC^-*R.irregularis*^ treatments, sagittain B (belonging to flavonol glycosides) showed the highest negative binding affinity (i.e. −9.46 Kcal·mol^−1^ with NodD1 and − 9.39 Kcal·mol^−1^ with NodD2) (Fig. 7F). It was only found in the RC*^M.truncatula^*/HC^+*R.irregularis*^ treatment and exhibited the highest integrated peak area simultaneously (Supplemental dataset 1). Meanwhile, NodD3 also plays a vital role in symbiotic signaling, particularly under flavonoid-independent conditions [38]. It regulates the expression of symbiotic genes through SyrM, thereby influencing the symbiotic process [38, 42]. This flavonoid-independent role explains the high expression of *nodD3* at 0 h (Fig. 6C) in our study, followed by a rapid decrease due to the subsequent upregulation of *nodD2* (Fig. 6B). Even though NodD3 does not require exogenous flavonoids for *nod* gene activation, the molecular docking analysis showed a high negative binding affinity for sagittain B (i.e. −9.08 Kcal·mol^−1^) (Supplementary 2). Thus, we could conclude that although flavonoids are less essential for NodD3, they could still act as signaling molecules to bind with NodD3 and modulate its interaction with other NodD proteins when sufficient external flavonoids are present. Moreover, NodD2 primarily induces *nod* gene expression and can compensate for the lack of NodD1 activity [44]. Our results supported this, showing that when *S. meliloti* was plated on the medium containing AM fungal hyphae, NodD1 was not rapidly activated, as evidenced by the lack of early (0, 2, and 4 h) *nodD1* gene expression upregulation (Fig. 6A). Instead, NodD2 quickly responded to external flavonoid signals, activating *nodD2* gene expression to compensate for the delayed NodD1 response, thereby initiating the downstream nodulation process (Fig. 6B). It has been reported that *S. meliloti* is not attracted to flavonoids found in alfalfa seed exudates [45]. However, that study was restricted to four exogenous flavonoids (hyperoside, luteolin, luteolin-7-glucoside, and chrysoeriol), which do not overlap with the eight flavonoids detected solely in the RC*^M.truncatula^*/HC^+*R.irregularis*^ treatment in our study. Therefore, their conclusion could not be extended to our results. Moreover, we performed a molecular docking analysis of the four flavonoids mentioned in previous study [45] with NodD1, NodD2, and NodD3 and observed that their most stable binding affinities were − 8.36, −8.37, and − 8.32 Kcal·mol^−1^, respectively (Supplementary 2, Fig. S16, S18, S20), which were much lower than the −9.46 and − 9.36 Kcal·mol^−1^ observed in our study. Based on these findings, we conclude that the distinct regulatory mechanisms of the NodD proteins underscore the complexity of the symbiotic signaling network in *S. meliloti*, where NodD1 and NodD2 are central to the initial flavonoid-mediated activation of *nod* genes.

Our *in vitro* data support that *S. meliloti* uses ERM exudates for catabolism and growth [46], forming biofilm-like multilayers on hyphae surfaces and spores, suggesting that *R. irregularis* provides nutrients for *S. meliloti* reproduction. Without ERM exudates, growth was significantly limited, whereas exudates from hyphae connected to *M. truncatula* or *S. tuberosum* promoted sigmoidal growth, with faster growth observed from *M. truncatula* hyphae exudates. This suggests that hyphae connected to *M. truncatula* release not only nutrients but also signals that may enhance *S. meliloti* growth, extending microbial perception beyond the rhizosphere. These observations need to be extended to other AM fungal species but *S. meliloti* seems to behave as a true fungiphile well-adapted to live in the hyphosphere of AM fungi.

In the migration experiment (Fig. 4A), *S. meliloti* preferentially migrated to the RC, indicating attraction by signals from hyphae connected to *M. truncatula*. Analysis of exudates from these hyphae suggests some of these signals may be the eight unique flavonoids detected in the RC*^M.truncatula^*/HC^+*R.irregularis*^ treatment. *S. meliloti* on the hyphae surface also stimulated cytoplasmic flow within the hyphae, suggesting that unknown molecules from *S. meliloti* enhance *R. irregularis* cytoplasmic flow. In the presence of rhizobia, flow speed was 10.78 μm·s^−1^, compared to 6.01 μm·s^−1^ without rhizobia, and 5.87 μm·s^−1^ with PBS. A higher resource allocation is therefore expected in presence of rhizobia, potentially stimulating the bacterial growth.

Mycelial networks, which are hydrophilic, expedite bacterial dispersal by providing continuous bacterial films around their hyphae [47–49]. The ERM can interconnect plants of the same or different species, forming CMNs [50–52]. These networks allow plants to receive warning signals and potentially exchange nutrients [53, 54]. Our study showed that *S. meliloti* inoculated on hyphae linking *M. truncatula* to *S. tuberosum* preferentially migrate toward *M. truncatula* (Fig. 4B), likely due to a gradient of chemoattractant signals (e.g. flavonoids) from *M. truncatula* connected hyphae, explaining slower migration towards *S. tuberosum*. This suggests that signal molecule concentration plays a crucial role in attracting *S. meliloti*, affecting its migration within the CMN. We detected eight unique flavonoids in the RC*^M.truncatula^*/HC*^+R.irregularis^* treatment. Their effects on *nod* gene expression and preferential migration towards *M. truncatula* suggest these flavonoids may drive *S. meliloti*’s selective preference. Previous studies support this, demonstrating that chemotaxis response to root exudates initiates rhizobia recruitment and root interactions [55, 56].

Flavonoids, also well-known as signaling molecules in AM symbiosis [57], were shown in this study to be transported by AM fungal hyphae, enabling the stimulation of rhizobia at distances far beyond the rhizosphere, thereby suggesting a mechanism for rhizobial migration and potential symbiosis establishment with legumes. Although our *in vitro* experiments provided initial evidence of rhizobial transport via AM fungal hyphae, the quadri-compartment pot experiment in the greenhouse, using a sand-vermiculite substrate, further demonstrated that this transport also occurs under more soil-like conditions, facilitating long-distance rhizobial dispersal and nodule formation. Hyphae could be a preferential route for long-distance *S. meliloti* migration, as shown in the greenhouse experiment. *S. meliloti* placed in the CC migrated toward *M. truncatula* connected by hyphae (approx. 15 cm). No nodules were observed without hyphae connections. Thus, hyphae likely serve as conduits, with their exudates acting as chemoattractants by providing nutrients and signaling molecules that help rhizobia sense compatible host plants. In addition, hyphae function as conduits, facilitating the rapid migration of rhizobia through soil to the roots, resulting in nodulation. Although ambient rhizobial levels in bulk soil are generally sufficient for nodulation of endemic legumes, their spatial heterogeneity can result in microsites with suboptimal densities, potentially limiting nodulation efficiency [58–63]. AM fungal hyphae can enhance rhizobial dispersal towards roots, especially in heterogeneous environments, aiding rapid colonization of new roots [64–67]. This dispersal mechanism may be particularly beneficial under natural ecosystem conditions where rhizobial distributions are uneven and competition in the rhizosphere is intense [7, 23, 68–70]. Our results suggest that, without being the only pathway, AM hyphae constitute an additional means of root acquisition of rhizobia, which could confer advantages in specific environmental scenarios. Further research needs to be conducted to validate these findings across different soil conditions and ecosystems.

## Conclusion

Root exudates can mediate the short-distance recruitment of rhizobia. For long distances (several cm) water mass flow [9] or other vectors [7] are often mentioned. Our study suggested that the ERM of AM fungi can serve as a route for rhizobia to migrate from long distance to the root, therefore representing an indirect pathway to initiate legume-rhizobia interaction. Given that all legumes are host to AM fungi, that hyphae are hospitable microhabitats for rhizobia and that AM fungal mycelium vastly extend in soil (from 82 to 111 m·cm^−3^ in prairie and 52 to 81 m·cm^−3^ in ungrazed pasture [12]), these below-ground fungi can be considered as important contributors in rhizobia dispersal in the soil. In addition to nutrients provision, the hyphae release signaling chemoattractant molecules facilitating the acquisition and movement of rhizobia to the legume. Our study supports the combined application of AM fungi and rhizobia to improve the efficiency of nitrogen fixation in legume crops. It also raises the hypothesis that agricultural practices that disturb the mycelial networks of AM fungi (e.g. physical disturbances) could indirectly influence rhizobia-legume interactions, particularly in soils where rhizobial density is low and/or in the absence of rhizobia seed coating, a relatively common agricultural practice, although further research is needed to explore these effects in concrete agronomic contexts. (Fig. 7).

## Supplementary Material

Supplementary_1_wrae185

Supplementary_2_wrae185

Video_S1_wrae185

Video_S2_wrae185

Supplemental_dataset_1_wrae185

## Data Availability

The authors declare that materials described in the manuscript, including all relevant raw data, will be freely available to any researcher wishing to use them for non-commercial purposes.
