# Supplementary material for: Arbuscular mycorrhizal hyphae facilitate rhizobia dispersal and nodulation in legumes
Source: ISME J. 2024 Sep 26;18(1):wrae185. doi: 10.1093/ismejo/wrae185 (PMC11520417; doi:10.1093/ismejo/wrae185)
Supplement: Supplementary_1_wrae185 [file supplementary_1_wrae185.docx]

# Supplementary Information

## Materials and methods

### Biological material

***Rhizophagus irregularis* MUCL 41833 –** The arbuscular mycorrhizal (AM) fungus *R. irregularis* (Blaszk., Wubet, Renker & Buscot) C. Walker & A. Schüßler comb. nov. MUCL 41833 was sourced from the Glomeromycota *in vitro* collection (GINCO, Belgium). This AM fungus was maintained *in vitro* using Ri T-DNA transformed roots of carrot (*Daucus carota* L.) clone G3. The culture was sustained in bi-compartmented Petri plates (90 mm in diameter), which were filled with sterilized (121°C for 15 min) Modified Strullu-Romand (MSR) medium [1], as described by Cranenbrouck et al. [2]. The plates were incubated in the dark at 27°C until needed for experimentation.

***Sinorhizobium meliloti* 2011 pHC60-GFP –** The bacterium *S. meliloti* 2011 pHC60-GFP [3] was provided by the VIB-UGent Center for Plant Systems Biology, Ghent University (Belgium). It was cultured at 28°C in yeast extract broth (YEB) medium, with tetracycline added at a concentration of 5 μg·mL^–1^. Prior to its use, the tetracycline solution was filtered using an Acrodisc Syringe Filter (0.2 μm Supor Membrane, Pall Corporation, New York, USA).

***Medicago truncatula* L., cv. Jemalong A17 –** Seeds of *M. truncatula* L., cv. Jemalong A17 were supplied by the South Australian Research and Development Institute (SARDI, Australia). These seeds underwent surface disinfection by immersion in an 8% active chloride bleach solution for 15 min, followed by three rinses with sterilized deionized water (121°C for 15 min), as per the protocol by Dupré de Boulois et al. [4]. Germination took place in Petri plates (90 mm in diameter, 10 seeds per plate) containing 40 mL of sterilized solid MSR medium, and the plates were incubated at 20°C in the dark for 7 d in an incubator (WTB binder-78532 TUTTLINGEN, Germany).

***Plantago lanceolata* L. –** Seeds of *P. lanceolata* L. were sourced from ECOSEM (Corroy-le-Grand, Belgium). The disinfection and germination processes were identical to those used for *M. truncatula*, ensuring consistency and sterility.

***Solanum tuberosum* L. cv Bintje –** *In vitro* propagated plantlets of *S. tuberosum* L. cv Bintje were supplied by the Station de Haute Belgique in Libramont, Belgium. Nodal cuttings were cultured in sterile culture boxes (80 mm diameter, 110 mm height, 10 cuttings per box) filled with 80 mL of 4.412 g·L^–1^ Murashige and Skoog (MS) medium [5], supplemented with 20 g·L^–1^ sucrose, 3 g·L^–1^ Phytagel (Sigma-Aldrich Co.), with pH adjusted to 5.9 before sterilization (121°C for 15 min).

The plantlets were maintained in a growth chamber at 22°C, with 16 h of light per day under a photosynthetic photon flux (PPF) of 100 μmol·m^–2^·s^–1^, and relative humidity (RH) of 70%, as detailed by Voets et al. [6].

### Composition of the Modified-Strullu-Romand medium lacking sucrose and vitamins (MSR^min^):

(1) MSR^min0N^ with 0 mM N (KNO_3_ and Ca(NO_3_)_2_·4H_2_O were replaced by 75.2 mM KCl and 152 mM CaCl_2_·2H_2_O, respectively) and (2) MSR^min½N^ containing half the N concentration (1.99 mM) of the normal MSR^min^ medium (i.e., 37.6 mM of KNO_3_ and 76 mM of Ca(NO_3_)_2_·4H_2_O).

### Mycelium Donor Plant (MDP) *in vitro* culture system

Bi-compartmented Petri plates (90 mm diam.) were used to grow excised transformed carrot roots and *Rhizophagus irregularis* as detailed by St-Arnaud et al. [7]. Briefly, in the first compartment (i.e. the root compartment – RC), 25 mL MSR medium containing 3 g·L^–1^ Phytagel was added, while in the second compartment (i.e. the hyphal compartment – HC), 25 mL MSR^min^ medium containing 3 g·L^–1^ Phytagel was added. An excised transformed carrot root associated with *R. irregularis* was transferred in the RC and incubated in a growth chamber at 27°C in the dark. Roots crossing the plastic barrier and growing in the HC were regularly trimmed using sterile forceps. After 13 weeks, a profuse extraradical mycelium (ERM) was observed in the HC. Three holes, each with a diameter of approximately 2 mm, were created at even intervals on the side of the HC (Fig. S2). Three seven-days-old *M. truncatula* or *S. tuberosum* seedlings were transferred to the HC, with the roots on the surface of the medium and the shoot extending outside the system. The systems were then sealed with Parafilm (Pechiney, Plastic Packaging, Chicago, IL 60631, USA) and the openings plastered with sterilized (121°C for 15 min) silicon grease (VWR International, Belgium). The systems were subsequently wrapped with opaque plastic bags to keep the AM fungus and plant roots in the dark, while shoots developed under light conditions. The systems were transferred to a growth chamber at 22/18°C (day/night), 70% relative humidity, a photoperiod of 16 h·day^-1^ and an average photosynthetic photon flux of 225 µmol·m^–2^·s^–1^. After 2 weeks, the plants were colonized by the AM fungus and used for subsequent experiments.

### Flavonoids analysis in the HC colonized by *R. irregularis*

Six mL of MSR^min0N^ medium collected in each HC was extracted with 6 mL methanol at room temperature for 1 h, followed by evaporation to dryness under vacuum conditions. The dry methanol extracts were resolubilized in 100 µL cyclohexane and 100 µL Milli-Q water, vortexed thoroughly, and then centrifuged at 14,000 rpm. Eighty µL of the aqueous phase was filtered using a 96-well filter plate. Samples were subjected to Ultra Performance Liquid Chromatography High Resolution Mass Spectrometry (UPLC-HRMS) at the VIB Metabolomics Core Ghent (VIB-MCG). Ten µL of the sample was injected on a Waters Acquity UHPLC (Waters) device connected to a Synapt XS high-definition mass spectrometer (Waters). Chromatographic separation was carried out on an ACQUITY UPLC BEH C18 (150 x 2.1 mm; 1.7 μm) column (Waters) with the column temperature maintained at 40 °C. A gradient of two buffers was used for separation, i.e., buffer A (water + 0.1% formic acid, pH 3) and buffer B (acetonitrile + 0.1% formic acid, pH 3). Buffer A was decreased from 99% to 50% in 30 min, decreased to 30% in 30 to 35 min, and further decreased to 0% in 35 to 37 min. The flow rate was set to 0.35 mL·min^–1^. Electrospray Ionization (ESI) was applied, and the LockSpray ion source was operated in negative ionization mode under the following conditions: capillary voltage, 3 kV; reference capillary voltage, 2.5 kV; source temperature, 120°C; desolvation gas temperature, 550°C; desolvation gas flow, 800 L·h^–1^; and cone gas flow, 50 L·h^–1^. The collision energy for full MS scan was set at 4 eV. For data-dependent acquisition–tandem mass spectrometry (DDA-MS/MS), the low mass ramp was ramped between 6-20 eV, and the high mass ramp was ramped between 20-70 eV. The mass range was set from 50 to 1500 Da and scan time was set at 0.3 s. The N (greater than 99.5 %) was employed as desolvation and cone gas. Leucine-enkephalin (100 pg·μL^–1^ solubilized in water:acetonitrile 1:1 [v/v], with 0.1% formic acid) was used for the lock mass calibration, scanning every 0.75 min at a scan time of 0.3 s. Profile data was recorded through Masslynx (Waters). Data processing was performed with Progenesis QI software version 3.0 (Waters) for chromatogram alignment and compound ion detection. The detection limit was set at medium sensitivity with a minimum peak width of 0.04 min. 20,081 compound ions were detected and aligned to “PooledSample_02n”. The data was normalized to all compound ions. The following filters were applied to analyze the data: a minimum ion intensity of 1,000 counts in at least one sample group (resulting in 13,513 features) and an ANOVA *p*-value ≤ 0.05 (resulting in 11,662 features). Statistical analyses were performed on ArcSinh-transformed and pareto scaled ion intensities. Structural annotation of the significant features was attempted using MS-FINDER (*in silico* fragmentation) [8]. The following parameter settings were applied: formula prediction and structural elucidation by *in silico* fragmenter using all available local databases, cut off score for structural elucidation: 5, MS^1^ mass tolerance: 5 ppm, MS^2^ mass tolerance: 10 ppm, relative abundance cut off: 0.1%, LEWIS and SENIOR check: TRUE, element ration check: common range (99.7%), element selection: C, H, N, tree depth: 2.

### *S. meliloti* growth and gene expression analyze

At week 14, two mL liquid MSR^min0N^ was collected from the HC of each Petri plate per treatment and transferred to a 2.5 mL centrifuge tube, followed by rapid freezing in liquid N and subsequent storage at –80°C. This solution was used to study its effects on the growth of *S. meliloti* (Fig. S9). One hundred and eighty µL of liquid MSR^min0N^ from each Petri plate was distributed in two wells (90 µL per well). Each treatment (RC*^M.truncatula^*/HC^+^*^R.irregularis^*, RC*^M.truncatula^*/HC^–^*^R.irregularis^*, RC*^S.tuberosum^*/HC^+^*^R.irregularis^* and RC*^S.tuberosum^*/HC^–^*^R.irregularis^*) comprised 6 Petri plates, thus 12 wells in total. Twenty µL of a solution of *S. meliloti* at a concentration of 9 × 10^5^ CFU·mL^–1^ (OD_600_ = 0.1) was gently mixed to the liquid MSR^min0N^ in the wells. To avoid boundary effects, the wells with MSR^min0N^ and bacteria were located at the center of the microplate, whereas 200 µL of fresh MSR^min0N^ medium without bacteria were added to the surrounding wells (thus corresponding to 48 wells). The microplate was then placed in a Multiskan FC Microplate Photometer (Thermo Fisher Scientific Inc., Waltham, MA) and incubated at 30°C for 72 h. The absorbance at OD = 595 nm was recorded each hour to evaluate the bacterial growth in each well. To convert OD_595_ to CFU, 0.4 mL of *S. meliloti* in its logarithmic growth phase was introduced into 20 mL of YEB medium. This mixture was then incubated at 28°C during 4 h to reach a stationary phase. The culture at stationary phase was serially diluted at specified ratios (5, 10, 50, 100, and 200), utilizing PBS as the diluent. Following the calibration of the spectrophotometer with PBS, the absorbance at 595 nm was recorded for varying concentrations of the bacterial solution. Subsequently, 1 mL from each dilution was plated on YEB agar medium (15 g·L^–1^ agar) supplemented with 5 μg·mL^–1^ tetracycline and evenly spread using an L-shaped cell spreader (VWR). The Petri plates were incubated for 24 h at 28°C in a dark chamber, after which the CFUs of *S. meliloti* were counted visually. The number of bacterial colonies for each dilution was documented, and the average colony count for each dilution level was calculated. A standard curve correlating absorbance values (X-axis) to the CFU (Y-axis) was generated using Microsoft Excel. The conversion from OD_595_ to CFU was done using the derived formula:

$$CFU=\frac{OD value-0.0072}{0.1016}\times{10}^{6}$$

The complete genome sequence of *S. meliloti* 2011 can be accessed at the National Center for Biotechnology Information (NCBI) under GenBank assembly accession number NC_020528.1. Eight genes (i.e., *nodA*, *nodB*, *nodC*, *nodD1*, *nodD2*, *nodD3*, *nodI*, *nodJ*) from *S. meliloti* were selected. The primers for the selected genes and their subunits were designed using Primer 5 software. Detailed information regarding the primer sequences and selected genes are presented in Table S2. Total RNA was extracted from the frozen solution of *S. meliloti* using the RNeasy Mini Kit (Qiagen), following the manufacturer’s protocol, and subsequently treated with the TURBO DNAfree Kit (Ambion) to eliminate possible DNA contamination. To generate single-stranded cDNA, reverse transcription was conducted using the Transcriptor High Fidelity cDNA Synthesis Kit (Roche) with 1 μL random primer. The reaction comprised the following steps: incubation at 65°C for 10 min, 55°C for 20 min, and 85°C for 5 min. The final volume of the cDNA was 20 μL and was diluted to 100 μL. The qRT-PCR was conducted using the LightCycler 96 Real-Time PCR System (Roche). The reaction mixture consisted of 2.5 μL cDNA, 5 μL 2 × FastStart Essential DNA Green Master (Roche), and 0.5 μL of each primer (5 μM), in a total reaction volume of 10 μL. The PCR was performed according to the following program: initial denaturation at 95°C for 60 s, followed by 45 cycles at 95°C for 10 s, 60°C for 10 s, and 72°C for 10 s. The run ended with a melting curve analysis to avoid nonspecific PCR products. The Ct values and baseline range were determined using the LightCycler 96 software. The relative quantification was evaluated using the 2^−ΔΔCT^ method [9], with *SMc00128* serving as the housekeeping gene. The transcript levels were normalized against the expression value in the RC*^S.tuberosum^*/HC^–^*^R.irregularis^* treatment.

### *S. meliloti* migration along AM fungal hyphae and cytoplasmic flow velocity within hyphae

At week 9, a profuse ERM development was observed in the RC and on the slope in the HC. At that time, 15 mL of MSR^min0N^ medium solidified with 3 g·L^–1^ Phytagel was added in the HC. At week 13, approximately 80% of the surface of the HC was covered with an extensive ERM.

***S. meliloti migration along hyphae –*** In each Petri plate, three hyphae, with active cytoplasmic/protoplasmic flow, developing on the surface of the HC and separated from each other by several mm, were individually inoculated with one drop of 1 µL of *S. meliloti* at a concentration of 9 × 10^5^ CFU·mL^–1^ (OD_600_ = 0.1). Six Petri plates (i.e., replicates) were considered per treatment. The speed of colonization of the hyphae surface was quantified by measuring the distance traveled by *S. meliloti* along the hyphae from the point of inoculation divided by the time post inoculation (i.e., after 2, 4, 6, 12 and 24 h).

***Velocity of cytoplasmic/protoplasmic flow in hyphae –*** Another set of 18 Petri plates were used to evaluate cytoplasmic/protoplasmic flow within the hyphae. Three hyphae developing on the surface of the HC and separated from each other by several mm were each inoculated with one drop of 1 µL of *S. meliloti* at a concentration of 9 × 10^5^ CFU·mL^–1^ (OD_600_ = 0.1) and cytoplasmic/protoplasmic flow within the hyphae measured. Two control treatments were included under strictly identical conditions: (1) hyphae inoculated with PBS and (2) non-inoculated hyphae. Six Petri plates (i.e., replicates) were considered per treatment.

***Image and video analysis –*** Images and videos were acquired using an Echo Revolve RVL2-K microscope (<https://discover-echo.com/revolve/>) ensuring at least a 1/4 overlap between adjacent images. The microscope’s software provided the automatic mode for all parameters (e.g. brightness, contrast and saturation, etc.), and the magnification was set at 4×. The captured images were stitched together using Fiji’s Stitching plugin [10]. The migration movies of *S. meliloti* along the hyphae was recorded via the 20× objective lens and measured using the Analyze menu’s Measure tool in Fiji [10]. The Measure tool was also used to quantify the thickness of the bacterial film around hyphae. To calculate the flow velocity in the hyphae, we extracted static frames at different time points from the recorded videos, and then measured the distance traveled by particles within hyphae between two intervals of time. Six measurements were recorded for one video captured from a single Petri plate and six Petri plates were considered in total (i.e. six replicates) and the average value was calculated.

### *In vitro* and greenhouse experimental designs with legumes linked to non-legumes by a CMN for mycelia-based migration assay of *S. meliloti*

***In vitro experimental design –*** Only three compartments of a quadri-compartmented (12.4 × 8.5 cm) Petri plate (modified from Voets et al. [11]) was used (Fig. 4a; Fig. S4), two RC were separated by a plastic barrier from a HC. The RCs received 25 mL of MSR^min½N^ medium, and the HC 25 mL of MSR^min0N^ medium. The mycorrhizal plants in the RCs were prepared from the MDP *in vitro* cultivation system (Fig. S2). Five mL of MSR^min½N^ medium cooled to 40 °C was added each week in the RCs to keep medium at an adequate level. At week 13, the ERM crossed the partition wall separating the RCs from the HC and developed profusely in the HC. A significant number of hyphae connected in the HC creating a CMN between both plants. Three single hyphae per system developing on the surface of the MSR^min0N^ medium and clearly connecting the plants in both RCs were inoculated with one drop of 1 µL of bacterial suspension at a concentration of 9 × 10^5^ CFU·mL^–1^ (OD_600_ = 0.1). Inoculation was done on the hyphae in the middle of the HC (Fig. 4a). The distance moved by *S. meliloti* in the direction of *M. truncatula* or *S. tuberosum* as well as the cytoplasmic flow velocity in the hyphae and thickness of the bacterial film around hyphae were measured as described above. Three hyphae of six independent replicates (i.e., quadri-compartmented Petri plates) were considered.

***Greenhouse experimental design –*** A quadri-compartmented pot system was designed comprising a central compartment (CC, 0.3 L) connected to three satellite compartments (0.3 L each) with plastic pipes (PVC pipe, 1.7 cm diameter × 10 cm length) (Fig. 5a; Fig. S7). The pipes were perforated in the upper part with six holes (6 mm diam.) separated from each other by 1 cm. The holes were used to collect the substrate (including the hyphae and bacteria). *P. lanceolata* was planted in the CC, and the three satellite compartments with *M. truncatula* associated (*M. truncatula*^+^*^R.i.^*) or not (*M. truncatula*^–^*^R.i.^*) to *R. irregularis* and *P. lanceolata* not associated to *R. irregularis* (*P. lanceolate*^–^*^R.i.^*). The *M. truncatula*^+^*^R.i.^* satellite compartment received 5 g of *R. irregularis* inoculum and was connected to the CC *via* a pipe sealed at both extremities with a 41 μm mesh to allow only hyphae and not the roots to proliferate in the tube. The *M. truncatula*^–^*^R.i.^* and *P. lanceolate*^–^*^R.i.^* satellite compartments were not inoculated by the AM fungus and were connected to the CC with the pipes sealed at both extremities with 5 μm mesh to prevent the crossing of hyphae and roots coming from the other compartments. *P. lanceolata* was planted in the CC only to check whether the hyphae from the *M. truncatula*^+^*^R.i.^* reached the CC. Once this was evidenced by root colonization assessment [12], it indicates that the AM fungi hyphae from the *M. truncatula*^+^*^R.i.^* treatment had reached the CC through the pipes. *P. lanceolata* in the CC were then removed, and after another 4 weeks (to allow mycelium regrowth in the CC), *S. meliloti* (5 mL of 9 × 10^6^ CFU·mL^–1^ - OD_600_ = 1) was inoculated in the CC using a 5 mL NORM-JECT silicone-free Luer Lock Syringe (Henke-Sass Wolf) and substrate samples were collected from the pipes of 5 randomly selected pot systems after 1, 3, 5, 7, and 9 d to evaluate *S. meliloti* migration. Samples were collected using 1 mL pipette tips cut at 3 cm length from the extremity. The tips were inserted into each hole of the pipes and connected to a 1.5 mL centrifuge tube to collect the sample. The pipes were inverted and tapped to allow the substrate samples to fall into the 1.5 mL centrifuge tubes. The substrate samples in the pipes were subsequently weighed. One mL of PBS was further added to each centrifuge tube and the tubes were vortexed on an MS1 shaker (IKA, Germany) at 1000 rpm for 20 s to extract the bacteria. A 20 μL suspension was retrieved from the aforementioned centrifuge tube and mixed with 180 μL of PBS to achieve a 10-fold dilution. For the growth of *S. meliloti*, a 20 μL volume of the diluted solution was added to YEB solid medium (15 g·L^–1^ agar) supplemented with 5 μg·mL^–1^ of tetracycline. The mixture was then spread using an L-Shape Cell Spreader (VWR). Bacteria were cultured for 48 h in a dark chamber at 28 °C. Then, the number of CFUs of *S. meliloti* were counted in each Petri plate using the Echo Revolve RVL2-K microscope (<https://discover-echo.com/revolve/>) and converted into CFUs per g of substrate sample.

Fresh roots were cut into 1 cm length sections and put in 50 mL centrifuge tubes for AM fungal root colonization assessment. Root staining and counting of root colonization were performed using the methods described by Walker [13] and McGonigle et al. [12], respectively.

The hyphal length in the pipes was measured using the modified method described by Bethlenfalvay and Ames [14]. Five grams of fresh substrate samples was placed in a 50 mL centrifuge tube, followed by the addition of 10 mL of 0.1 mol·L^–1^ PBS (pH 7.8). After gently shaking, 8 mL of the sample solution was transferred to a 10 mL centrifuge tube. Subsequently, 2 mL of 2% ink (Parker Blue Ink, United States) in 1% HCl was added to the tube, mixed gently, and incubated at 70°C for 20 min. The solution was then cooled to room temperature, and 1 mL was extracted for microscopic examination using an Olympus BH2-RFCA microscope (Japan) at 10× magnification.

Two-, four-, and eight-weeks post-inoculation with *S. meliloti*, five randomly selected microcosm systems were harvested and the total number of nodules in each plant of the three satellite compartments was evaluated under a stereomicroscope (Olympus-SZ61, Japan) at 0.67× magnification. In addition, nodule maturity was assessed based on color, with white representing immature and red signifying mature nodules.

## Result

### Expression of structural *nod* genes

we analyzed the expression of the structural *nod* genes. The expression of *nodA* was significantly higher in the RC*^M.truncatula^*/HC^+^*^R.irregularis^* treatment at 4, 6, 12, and 24 h compared to both controls and the RC*^S.tuberosum^*/HC^+^*^R.irregularis^* treatment (Fig. S5a). In the RC*^S.tuberosum^*/HC^+^*^R.irregularis^* treatment, *nodA* expression was significantly higher at 2, 4, 6, and 12 h compared to both controls (Fig. S5a). For *nodB*, the RC*^M.truncatula^*/HC^+^*^R.irregularis^* treatment showed significantly higher expression compared to both controls at all time points (Fig. S5b). In contrast, the RC*^S.tuberosum^*/HC^+^*^R.irregularis^* treatment had significantly higher *nodB* expression than the controls at 0, 2, and 6 h, but significantly lower at 12 h (Fig. S5b). When comparing both treatments, *nodB* expression was significantly higher in the RC*^M.truncatula^*/HC^+^*^R.irregularis^* treatment at 4, 6, 12, and 24 h, and significantly lower at 0 and 2 h, compared to the RC*^S.tuberosum^*/HC^+^*^R.irregularis^* treatment (Fig. S5b). The expression of *nodC* was significantly higher in both RC*^M.truncatula^*/HC^+^*^R.irregularis^* and RC*^S.tuberosum^*/HC^+^*^R.irregularis^* treatments compared to both controls at 0, 2, and 24 h (Fig. S5c). The *nodC* expression was not significantly different between the two treatments, except at 2 h where it was higher in the RC*^S.tuberosum^*/HC^+^*^R.irregularis^* treatment, and at 4, 12, and 24 h where it was higher in the RC*^M.truncatula^*/HC^+^*^R.irregularis^* (Fig. S5c). For *nodI*, both RC*^M.truncatula^*/HC^+^*^R.irregularis^* and RC*^S.tuberosum^*/HC^+^*^R.irregularis^* treatments showed significantly higher expression compared to both controls only at 24 h, with lower expression at all other time points, except 0 and 4 h (Fig. S5d). The expression of *nodJ* in the RC*^M.truncatula^*/HC^+^*^R.irregularis^* treatment was significantly higher compared to both controls at 2 and 4 h, and significantly lower at 6 and 12 h (Fig. S5e). In the RC*^S.tuberosum^*/HC^+^*^R.irregularis^* treatment, *nodJ* expression was significantly higher than both controls only at 2 h and significantly lower at all other time points, except at 24 h where there was no difference (Fig. S5e). Additionally, *nodJ* expression was significantly higher in the RC*^M.truncatula^*/HC^+^*^R.irregularis^* treatment at 0, 2, 4, and 6 h compared to the RC*^S.tuberosum^*/HC^+^*^R.irregularis^* treatment (Fig. S5e). Overall, the expression of most genes (*nodA*, *nodB*, *nodD1*, *nodD2* and *nodD3*) significantly increased after the 6^th^ h in the RC*^M.truncatula^*/HC^+^*^R.irregularis^* treatment suggesting that these genes play a crucial role throughout the regulatory process.


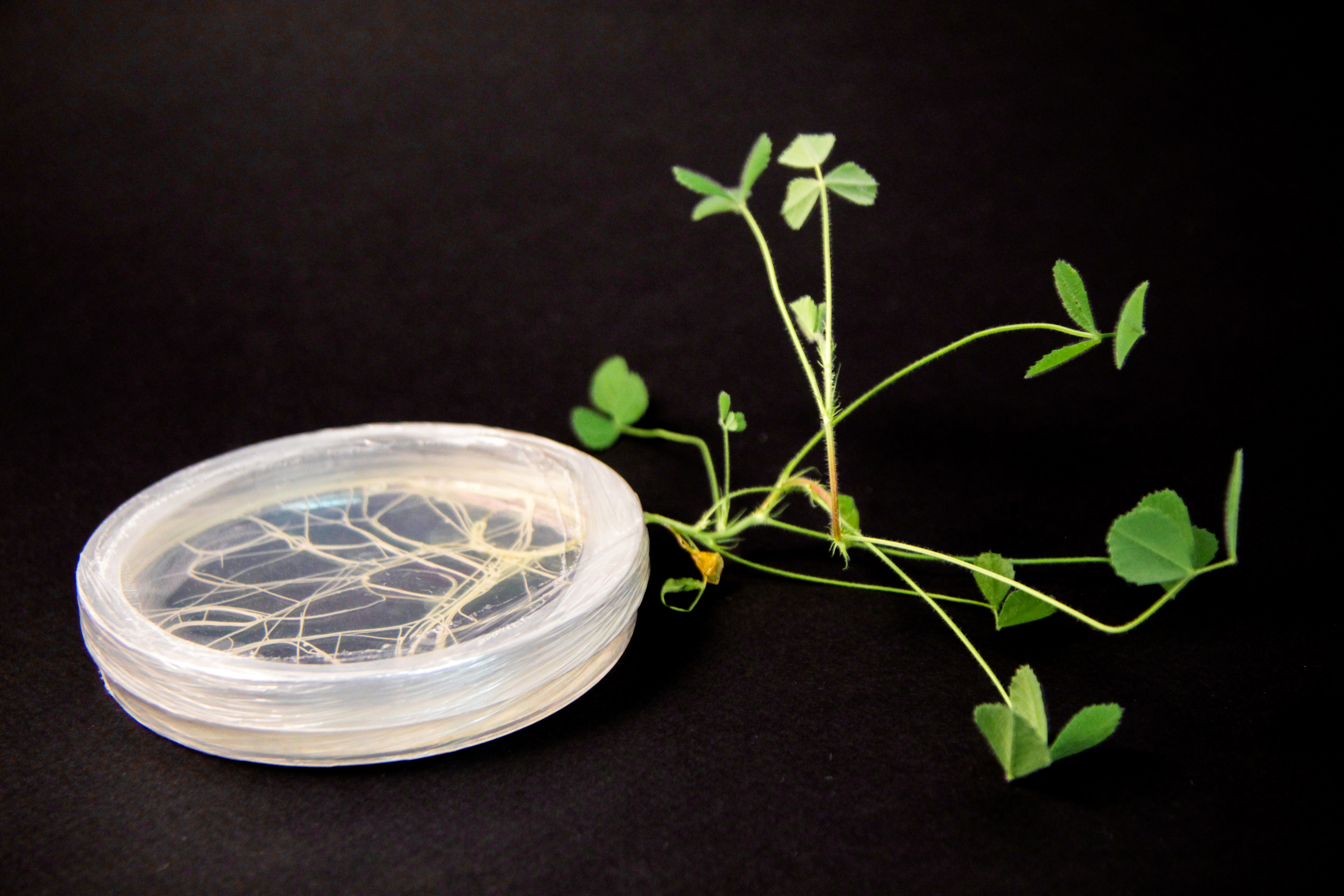


**Fig. S1** Picture of the mono-compartment autotrophic *in vitro* culture system with *Medicago truncatula*.


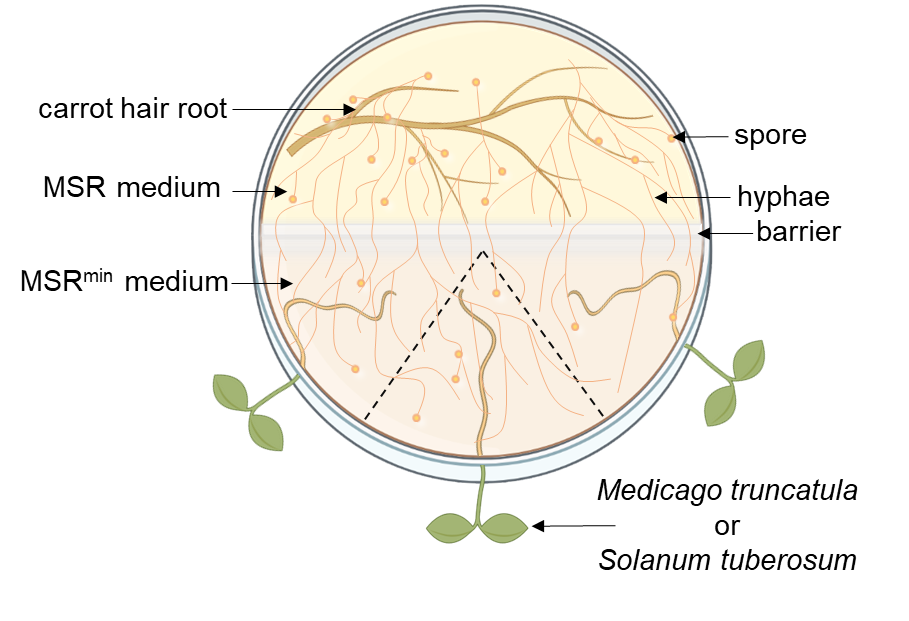


**Fig. S2** Schematic representation of the mycelium donor plant (MDP) *in vitro* culture system for the pre-colonization of plants.


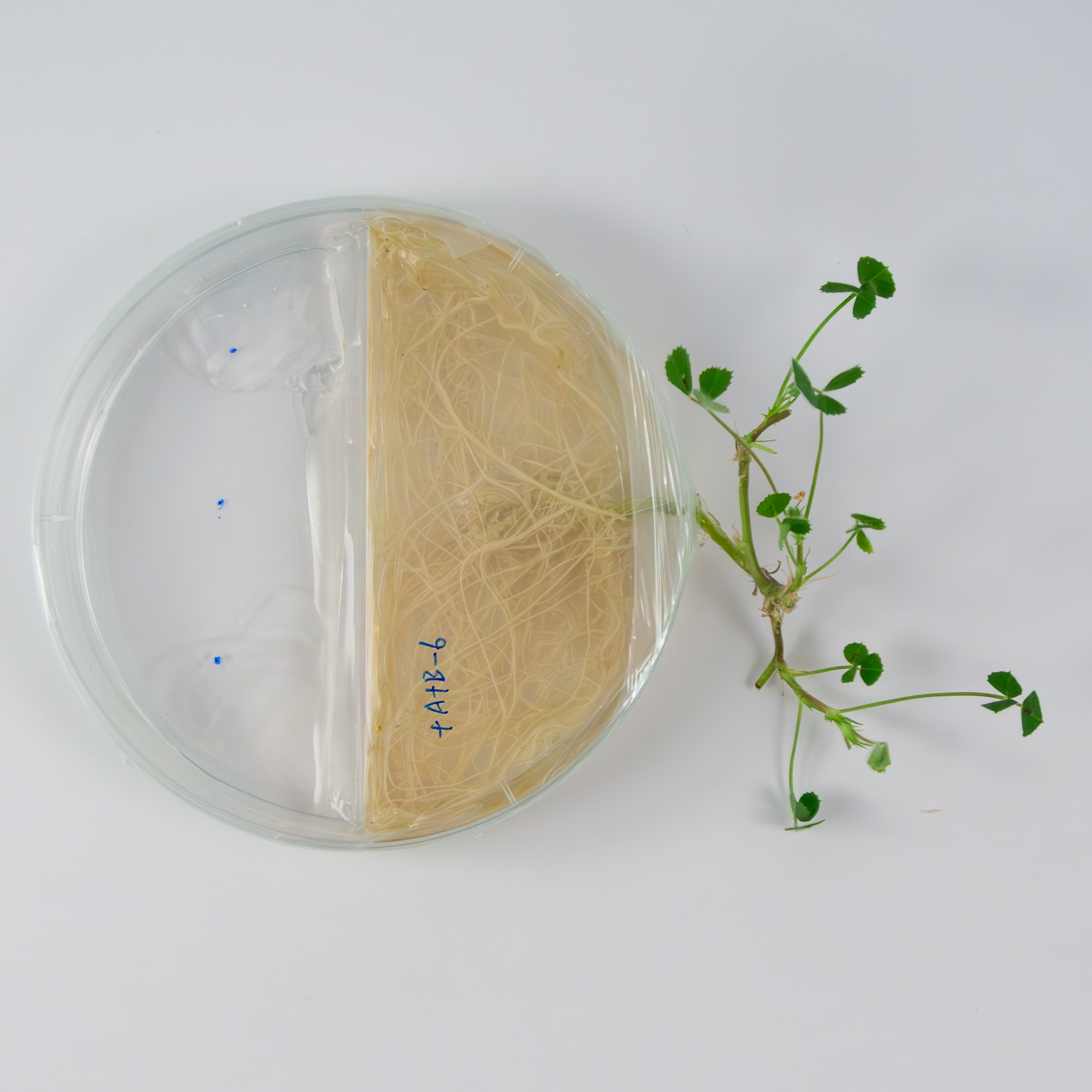


**Fig. S3** Picture of the bi-compartment autotrophic *in vitro* culture system with *M. truncatula*.


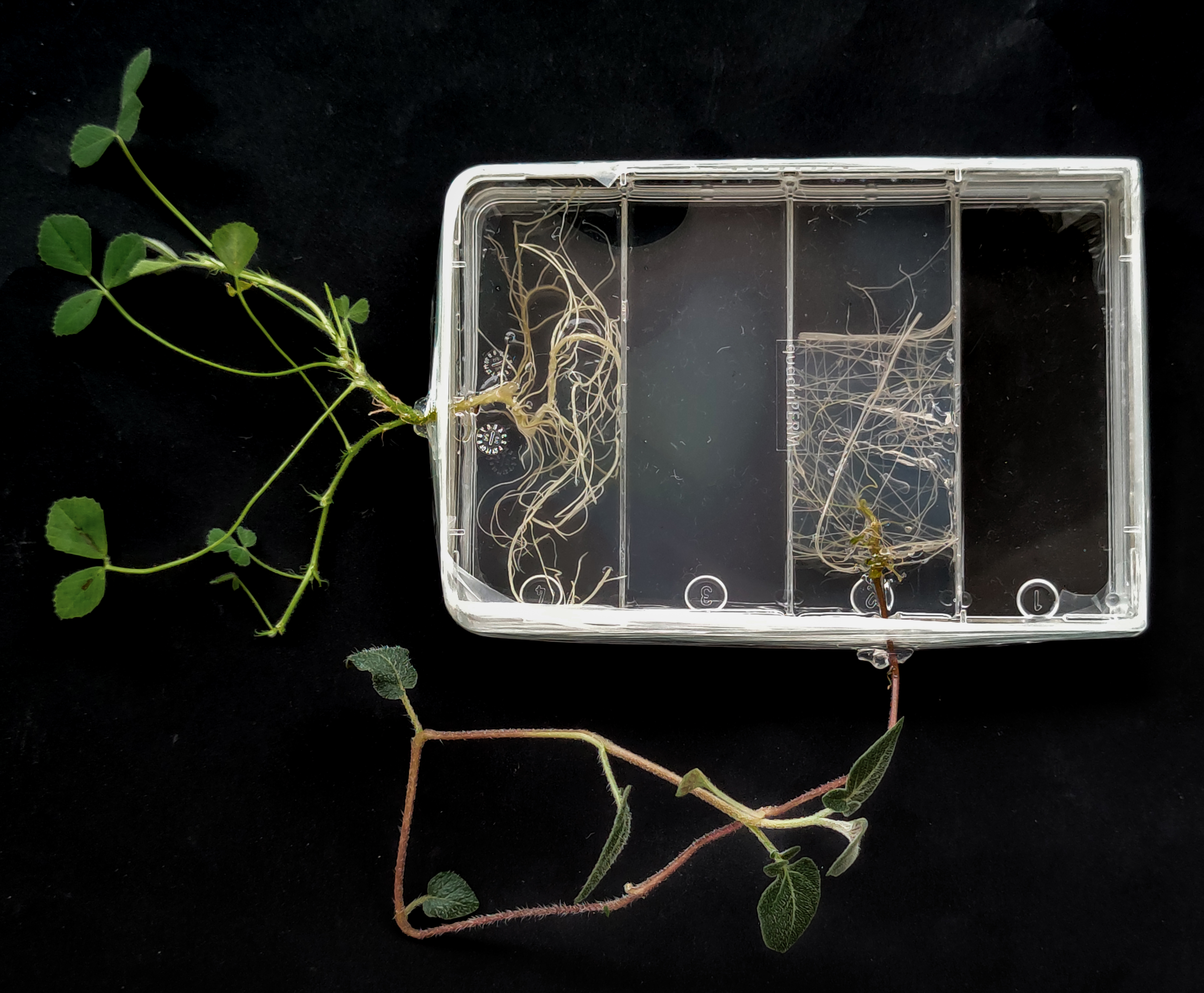


**Fig. S4** Picture of the quadri-compartmented autotrophic *in vitro* culture system.


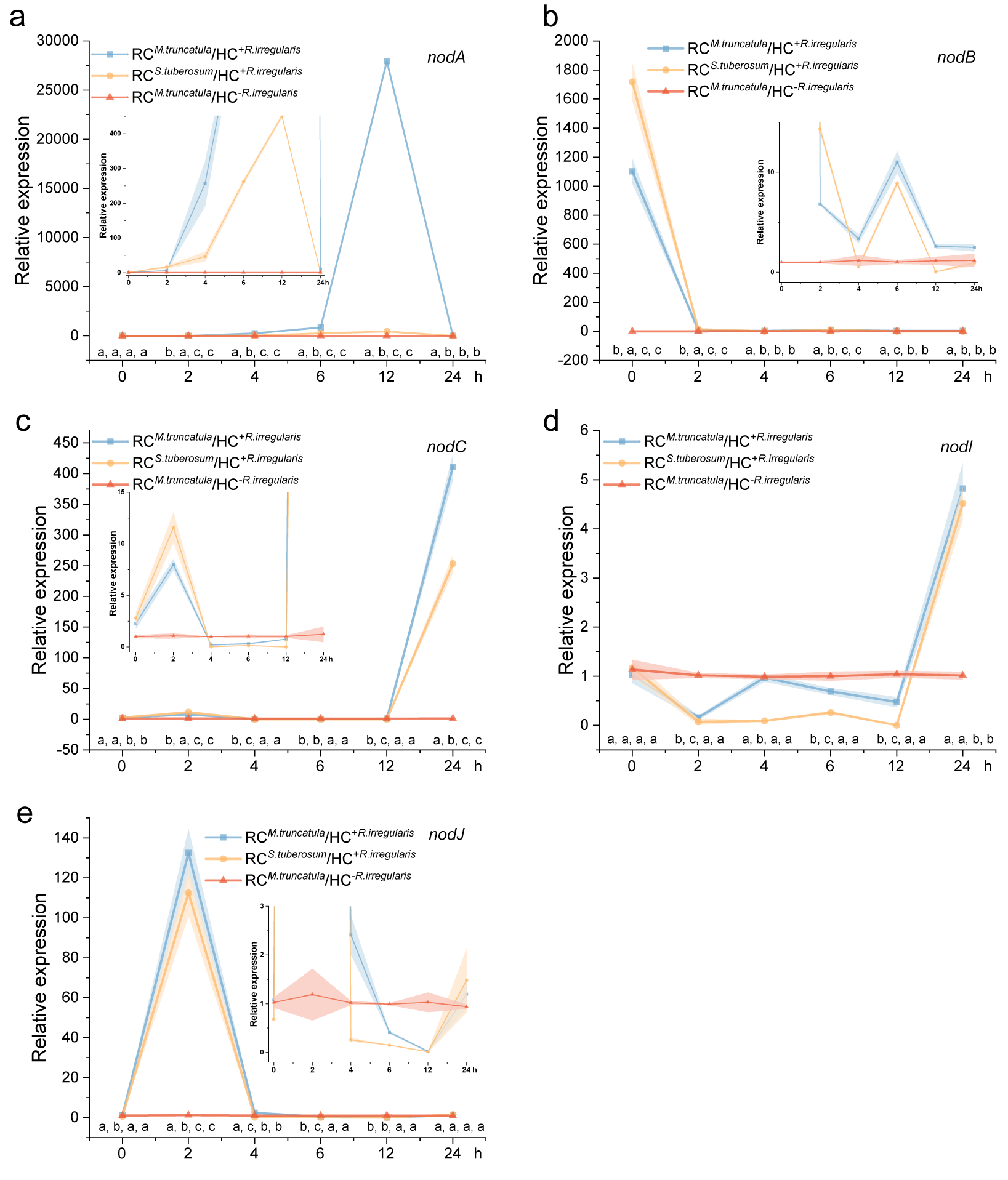


**Fig. S5** Impact of ERM exudates on the relative expressions of *nodA* **(a)**, *nodB* **(b)**, *nodC* **(c)**, *nodI* **(d)** and *nodJ* **(e)** in *S. meliloti* at 0, 2, 4, 6, 12, and 24 h after inoculation of the bacteria in the HC of RC*^M.truncatula^*/HC^+^*^R.irregularis^*, RC*^M.truncatula^*/HC^–^*^R.irregularis^*, RC*^S.tuberosum^*/HC^+^*^R.irregularis^* and RC*^S.tuberosum^*/HC^–^*^R.irregularis^* treatments. The results were normalized by comparing the transcripts to the relative expression value in RC*^S.tuberosum^*/HC^–^*^R.irregularis^* treatment (not shown in the figures). The light-colored background behind the lines on the figure represents the connecting error bars. Values are averages with SD. Data (n = 6) were analyzed by a one-way ANOVA followed by the Tukey post-hoc test (*p* ≤ 0.05). Different lowercase letters above each time point on the X-axis indicate significant differences between treatments at that time point, with the letters in sequence representing RC*^M.truncatula^*/HC^+^*^R.irregularis^*, RC*^M.truncatula^*/HC^–^*^R.irregularis^*, RC*^S.tuberosum^*/HC^+^*^R.irregularis^* and RC*^S.tuberosum^*/HC^–^*^R.irregularis^*. RC*^M.truncatula^*/HC^+^*^R.irregularis^*: with *M. truncatula* in the RC and hyphae in the HC; RC*^M.truncatula^*/HC^–^*^R.irregularis^*: with *M. truncatula* in the RC but no hyphae in the HC; RC*^S.tuberosum^*/HC^+^*^R.irregularis^*: with *S. tuberosum* in the RC and hyphae in the HC; RC*^S.tuberosum^*/HC^–^*^R.irregularis^*: with *S. tuberosum* in the RC but no hyphae in the HC.

**Fig. S6** The movement track for *S. meliloti* on the surface of ERM in the hyphal compartment (HC) with *M. truncatula* in the root compartment (RC) of bi-compartmental Petri plates after 12 h


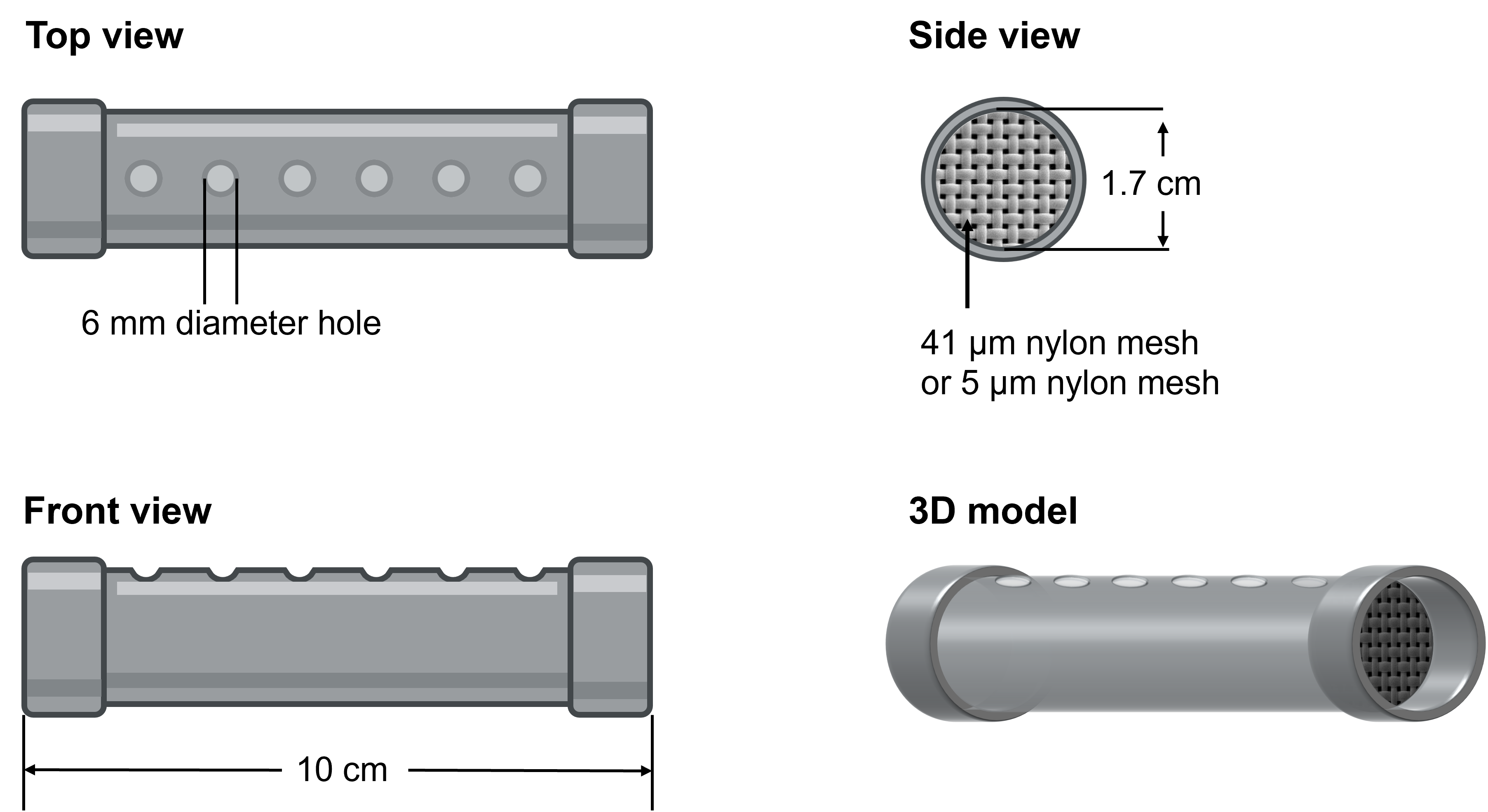


**Fig. S7** Schematic diagrams of the pipe from different perspectives.

**
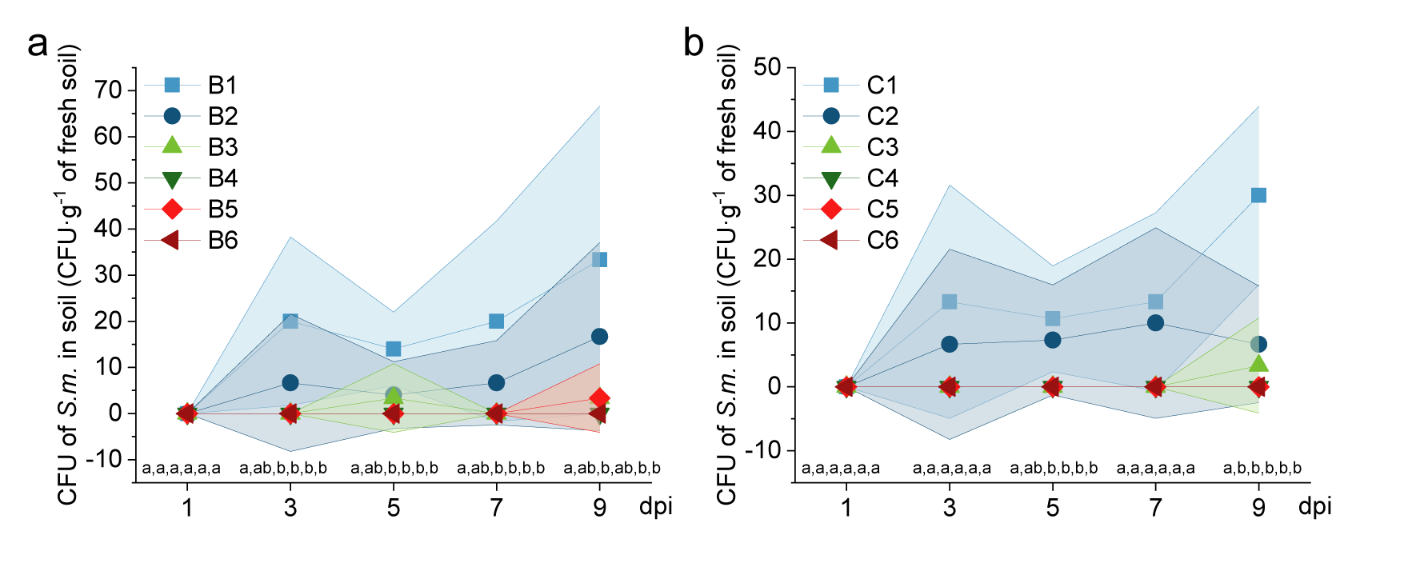
**

**Fig. S8** Concentration of *S. meliloti* (CFU·g^–1^ of fresh substrate) in the different holes in the pipes connecting the CC to the *M. truncatula*^–^*^R.i.^* (B1 to B6) **(a)** or *P. lanceolata*^–^*^R.i.^* (C1-C6) **(b)** at 1, 3, 5, 7, and 9 d post inoculation (dpi) after *S. meliloti* inoculation in the CC. Data (n = 5) are represented as means ± SD. The light-colored background behind the lines on the figure represents the connecting SD bars.


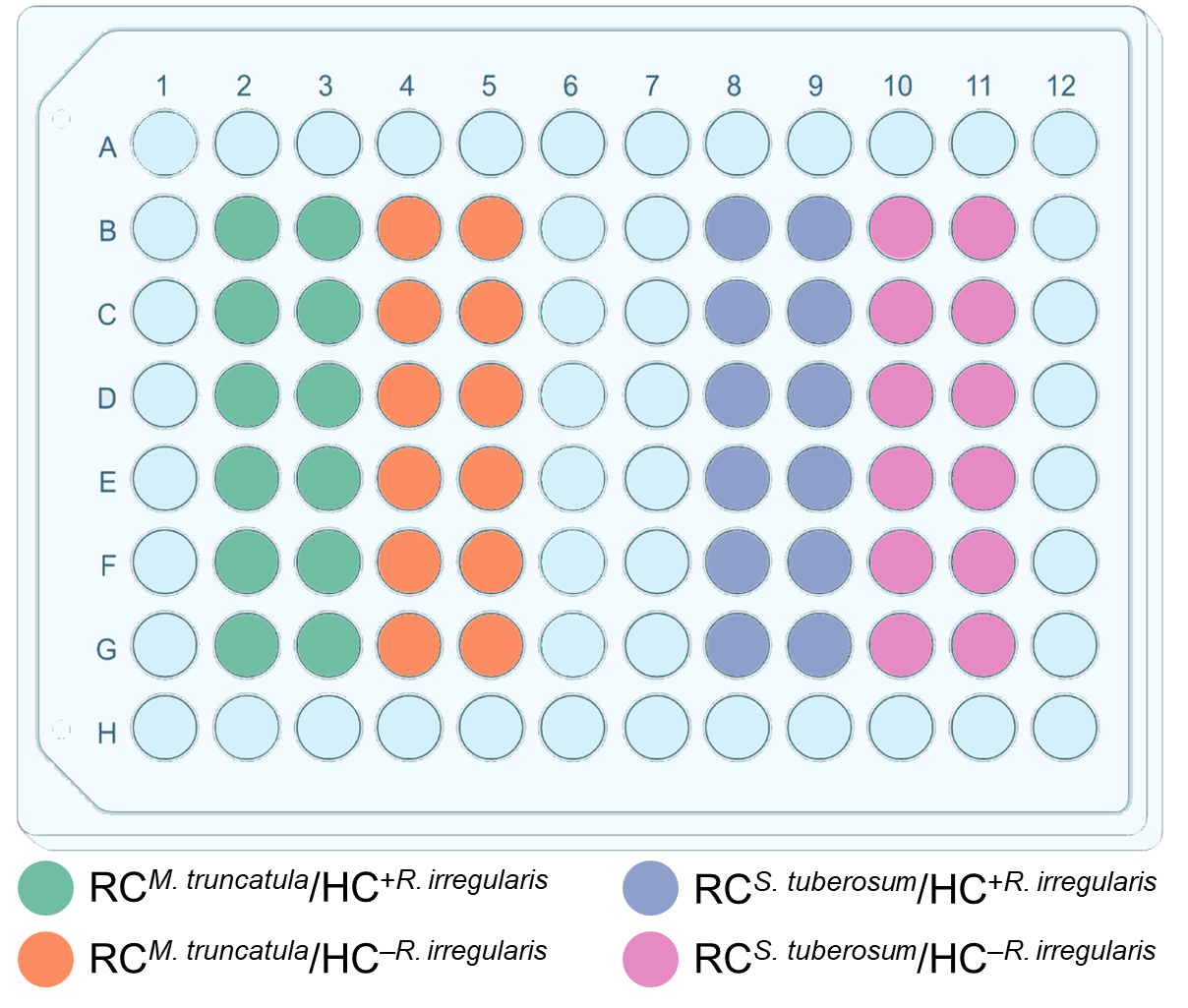


**Fig. S9** Layout of 96-wells plate culture of *S. meliloti*. Blue wells: 200 µL of fresh MSR^min0N^ medium without bacteria to avoid boundary effects. RC*^M.truncatula^*/HC^+^*^R.irregularis^* (green wells): with *Medicago truncatula* in the root compartment and hyphae in the hyphae compartment; RC*^M.truncatula^*/HC^–^*^R.irregularis^* (orange wells): with *M. truncatula* in the root compartment but no hyphae in the hyphae compartment; RC*^S.tuberosum^*/HC^+^*^R.irregularis^* (dark blue wells): with potato in the root compartment and hyphae in the hyphae compartment; RC*^S.tuberosum^*/HC^–^*^R.irregularis^* (pink wells): with potato in the root compartment but no hyphae in the hyphae compartment.

**Table S1** Root mycorrhizal colonization and soil mycelium density in the *in vivo* experiment.

| **Sampling date (Y-M-D)** | **Sampling place** | **Arbuscules (%)** | **Vesicles/spores (%)** | **Total (%)** | **Soil mycelium density (cm·g^–1^ of fresh soil)** |
| --- | --- | --- | --- | --- | --- |
| 2023-07-06 | CC | 30.3 ± 4.3 | 19.7 ± 4.5 | 82.5 ± 4.4 | -- |
| 2023-08-01 | *M. truncatula*^+^*^R.i.^* | 38.5 ± 2.8 | 18.8 ± 2.5 | 79.3 ± 1.3 | 69.1 ± 10.7 |
| 2023-08-01 | *M. truncatula*^–^*^R.i.^* | 0 ± 0 | 0 ± 0 | 0 ± 0 | 0 ± 0 |
| 2023-08-01 | *P. lanceolata*^–^*^R.i.^* | 0 ± 0 | 0 ± 0 | 0 ± 0 | 0 ± 0 |
| 2023-08-01 | CC | -- | -- | -- | 51.5 ± 6.5 |
| 2023-08-01 | *M. truncatula*^+^*^R.i.^* tube | -- | -- | -- | 73.3 ± 9.6 |
| 2023-08-01 | *M. truncatula*^–^*^R.i.^* tube | -- | -- | -- | 0 ± 0 |
| 2023-08-01 | *P. lanceolata*^–^*^R.i.^* tube | -- | -- | -- | 0 ± 0 |

**Note:** CC: central chamber; *M. truncatula*^+^*^R.i.^*: the chamber with *M. truncatula* and *R. irregularis*; *M. truncatula*^–^*^R.i.^*: the chamber with *M. truncatula* but not *R. irregularis*; *P. lanceolata*^–^*^R.i.^*: the chamber with *P. lanceolata* but not *R. irregularis*; *M. truncatula^+R.i.^* tube: the tube connectes *M. truncatula*^+^*^R.i.^* with the CC; *M. truncatula*^–^*^R.i.^* tube: the tube connectes *M. truncatula*^–^*^R.i.^* with the CC; *P. lanceolata*^–^*^R.i.^* tube: the tube connectes *P. lanceolata*^–^*^R.i.^* with the CC. Data (n = 6) are represented as means ± SD.

**Table S2** Gene-specific primer sequences used in this study.

| **Gene** | **Abbreviations in this study** | **GenBank accession number** | **Gene length (bp)** | **Primer sequence (5’-3’)** | **Product length (bp)** |
| --- | --- | --- | --- | --- | --- |
| Multispecies: nodulation N-acyltransferase NodA | *nodA* | WP_010967455 | 591 | F: GGTCGGGATAGCAAGCCACATG  R: CCAGTTCAGCCACAAGGAGATCAG | 80 |
| NodB chitooligosaccharide deacetylase (plasmid) | *nodB* | AGG70157 | 654 | F: CGGAAGGTCACGAAGTGGCTAAC  R: CTGAGGACAAGCGGCGATAATGG | 122 |
| NodC N-acetylglucosaminyltransferase (plasmid) | *nodC* | AGG70156 | 1281 | F: TCCAGAGGTCGGTGCGGTTATG  R: TTCGTTACAGGCAAGCCAATACTCC | 103 |
| NodD1 nod-box dependent transcriptional activator (plasmid) | *nodD1* | AGG70159 | 927 | F: CCACGCCTATTGTCGGGTACTAAC  R: CGGCAGCGGAGATGTGACTATG | 105 |
| NodD2 nod box-dependent transcription activator (plasmid) | *nodD2* | AGG70091 | 933 | F: GCCGCACACCAATCGTATAGCC  R: TGGACAGCCTCAGTGAACCAGAG | 120 |
| NodD3 nod box-dependent transcriptional activator (plasmid) | *nodD3* | AGG70144 | 942 | F: ACTCGATGCACTGATGACCAAACG  R: ATAGGAGCGTAGGCGAGCGATG | 103 |
| NodI ABC transporter, ATP-binding protein (plasmid) | *nodI* | AGG70155 | 1008 | F: GCGGCAAGACGATTCTCCTGAC  R: CTATCACCTGGCAACCGATCTTCTC | 147 |
| NodJ ABC transporter, permease (plasmid) | *nodJ* | AGG70154 | 789 | F: GGTGGAACTGGATTGCCGTGTG  R: CGCCCGACCATCATTCCTAAGC | 139 |
| Hypothetical protein, signal peptide | *SMc00128* | AGG73587 | 546 | F: TTGCGATCTTCGACAGCGG  R: GCAGTTCGACGAGCTGGATC | 111 |

**Video S1** Movement of *S. meliloti* on the surface of ERM in the hyphal compartment in the direction of the root compartment containing *M. truncatula* at 12 h following inoculation of the bacteria on the hyphae.

**Video S2** Velocity of cytoplasmic/protoplasmic flow in the ERM at 24 h following *S. meliloti* inoculation on the hyphae, under the bright field of view.

## References

1. Declerck S, Strullu DG, Plenchette C. Monoxenic culture of the intraradical forms of *Glomus* sp. isolated from a tropical ecosystem: a proposed methodology for germplasm collection. *Mycologia*. 1998; 90:579–585.

2. Declerck S, Strullu D-G, Fortin JA. In vitro culture of mycorrhizas. 2005. Springer, Berlin [etc.].

3. Cheng H-P, Walker GC. Succinoglycan Is Required for Initiation and Elongation of Infection Threads during Nodulation of Alfalfa by *Rhizobium meliloti*. *J Bacteriol*. 1998; 180:5183–5191.

4. De Boulois HD, Voets L, Delvaux B, Jakobsen I, Declerck S. Transport of radiocaesium by arbuscular mycorrhizal fungi to *Medicago truncatula* under *in vitro* conditions. *Environ Microbiol*. 2006; 8:1926–1934.

5. Murashige T, Skoog F. A Revised Medium for Rapid Growth and Bio Assays with Tobacco Tissue Cultures. *Physiol Plant*. 1962; 15:473–497.

6. Voets L, Dupré De Boulois H, Renard L, Strullu D-G, Declerck S. Development of an autotrophic culture system for the *in vitro* mycorrhization of potato plantlets. *FEMS Microbiol Lett*. 2005; 248:111–118.

7. St-Arnaud M, Hamel C, Vimard B, Caron M, Fortin JA. Enhanced hyphal growth and spore production of the arbuscular mycorrhizal fungus *Glomus intraradices* in an *in vitro* system in the absence of host roots. *Mycol Res*. 1996; 100:328–332.

8. Tsugawa H, Kind T, Nakabayashi R, Yukihira D, Tanaka W, Cajka T, et al. Hydrogen Rearrangement Rules: Computational MS/MS Fragmentation and Structure Elucidation Using MS-FINDER Software. *Anal Chem*. 2016; 88:7946–7958.

9. Livak KJ, Schmittgen TD. Analysis of Relative Gene Expression Data Using Real-Time Quantitative PCR and the 2^−ΔΔCT^ Method. *Methods*. 2001; 25:402–408.

10. Schindelin J, Arganda-Carreras I, Frise E, Kaynig V, Longair M, Pietzsch T, et al. Fiji: an open-source platform for biological-image analysis. *Nat Methods*. 2012; 9:676–682.

11. Voets L, De La Providencia IE, Declerck S. Glomeraceae and Gigasporaceae differ in their ability to form hyphal networks. *New Phytol*. 2006; 172:185–188.

12. McGONIGLE TP, Miller MH, Evans DG, Fairchild GL, Swan JA. A new method which gives an objective measure of colonization of roots by vesicular-arbuscular mycorrhizal fungi. *New Phytol*. 1990; 115:495–501.

13. Walker C. A simple blue staining technique for arbuscular mycorrhizal and other root-inhabiting fungi. *Inoculum*. 2005; 56:68–69.

14. Bethlenfalvay GJ, Ames RN. Comparison of two methods for quantifying extraradical mycelium of vesicular-arbuscular mycorrhizal fungi. *Soil Sci Soc Am J*. 1987; 51:834–837.
