# Supplementary material for: Arbuscular mycorrhizal hyphae facilitate rhizobia dispersal and nodulation in legumes
Source: ISME J. 2024 Sep 26;18(1):wrae185. doi: 10.1093/ismejo/wrae185 (PMC11520417; doi:10.1093/ismejo/wrae185)
Supplement: Supplementary_2_wrae185 [file supplementary_2_wrae185.docx]

# In silico binding studies

**Methods**

The in silico binding studies was made according to the protocol described in Bag et al. [1]. Fourteen flavonoids were considered (see Table S3) as well as three proteins from *Sinorhizobium meliloti*: NodD1 (UniProtKB-P03031), NodD2 (UniProtKB-P08719), and NodD3 (UniProtKB-P23190) from the UniProt Knowledgebase (<https://www.uniprot.org/help/uniprotkb>). The 3D structures of the flavonoids were obtained from PubChem ([https://pubchem.ncbi.nlm.nih.gov](https://pubchem.ncbi.nlm.nih.gov/)) as SDF files and subsequently converted into PDB files using PyMOL 3.0 (ref. 2). For flavonoids lacking available 3D structures in PubChem, the Chem3D (version 21.0.0) software was utilized to generate their 3D conformations. The 3D structures of the NodD proteins (NodD1, NodD2, and NodD3) were modeled using the SWISS-MODEL tool ([https://swissmodel.expasy.org](https://swissmodel.expasy.org/)) based on their respective FASTA sequences. Molecular docking studies were conducted using AutoDock 4.2 to form complexes between each NodD protein and each flavonoid, to determine their binding affinities (Kcal·mol^−1^) [3]. Additionally, LigPlot was used to identify the specific interacting residues within these complexes, providing further insight into the molecular interactions between the flavonoids and the NodD proteins [4].

**Table S3** The information of flavonoids in silico NodD1,2,3–ﬂavonoid binding studies

| **No.** | **Flavonoids** | **PubChem CID** | **m/z** |
| --- | --- | --- | --- |
| 1 | Luteolin-acetylhexuronide | 44258127 | 503.0830 |
| 2 | Sudachiin A | 73829938 | 521.1300 |
| 3 | Tetrahydroxy-tetramethoxyflavone-hexoside | 44260070 | 567.1355 |
| 4 | Isosakuranetin-hexosyl-deoxyhexose | 16760075 | 593.1875 |
| 5 | Taxifolin-deoxyhexosyl-oxanepentol | 163060888 | 597.1460 |
| 6 | Haploside C | 44260015 | 695.1832 |
| 7 | Sagittain B1 | 44258973 | 751.2116 |
| 8 | Sagittain B2 | 44258973 | 751.2117 |
| 9 | Trimethoxy-hydroxy-methyl-flavanone-dihexosyl-deoxyhexose | 72828014 | 813.2828 |
| 10 | Trihydroxy-flavanone-hexosyl-pentose | 42607913 | 565.1563 |
| 11 | Luteolin-7-glucoside | 5280637 | 447.0938 |
| 12 | Hyperoside | 5281643 | 303.0502 |
| 13 | Luteolin | 5280445 | 287.0543 |
| 14 | Chrysoeriol | 5280666 | 299.0553 |

**Fig. S10** In silico binding studies between NodD1 protein of *S. meliloti* and 10 flavonoids. Here NodD1–flavonoid complexes were arranged based on their binding affinities (the more negative the values, (Kcal·mol^−1^), the stronger the interactions). Each NodD1–flavonoid complex form hydrogen (green dotted line between amino acid and flavonoid) and hydrophobic (red half circles) bonds during NodD1–flavonoid interaction. The red-boxed NodD1–flavonoid indicates the flavonoid found exclusively in the RC*^S.tuberosum^*/HC^+^*^R.irregularis^* treatment. The central picture (inside the oval circle) predicts the binding sites of 9 flavonoids in NodD1 protein of *S. meliloti*. Individual figures are shown below. A: sagittain B1 and sagittain B2; B: isosakuranetin-hexosyl-deoxyhexose; C: luteolin-acetylhexuronide; D: taxifolin-deoxyhexosyl-oxanepentol; E: haploside C; F: sudachiin A; G: tetrahydroxy-tetramethoxyflavone-hexoside; H: trihydroxy-flavanone-hexosyl-pentose; I: trimethoxy-hydroxy-methyl-flavanone-dihexosyl-deoxyhexose. Sagittain B1 and Sagittain B2 were merged into a single flavonoid since their comparison results in the PubChem ([https://pubchem.ncbi.nlm.nih.gov](https://pubchem.ncbi.nlm.nih.gov/)) were identical. The figure was designed according to Bag et al. [1].

**Fig. S11** In silico binding studies of NodD1 protein of *S. meliloti* and 10 flavonoids. Each NodD1-flavonoid complex form hydrogen (green dotted line between amino acid and flavonoid, with distances) and hydrophobic (red half circles) bond during NodD1-flavonoid interaction. Sagittain B1 and Sagittain B2 were merged into a single flavonoid since their comparison results in the PubChem ([https://pubchem.ncbi.nlm.nih.gov](https://pubchem.ncbi.nlm.nih.gov/)) were identical.

**Fig. S12** In silico binding studies between NodD2 protein of *S. meliloti* and 10 flavonoids. Here NodD2–flavonoid complexes were arranged based on their binding affinities (the more negative the values, (Kcal·mol^−1^), the stronger the interactions). Each NodD2–flavonoid complex form hydrogen (green dotted line between amino acid and flavonoid) and hydrophobic (red half circles) bonds during NodD2–flavonoid interaction. The red-boxed NodD2–flavonoid indicates the flavonoid found exclusively in the RC*^S.tuberosum^*/HC^+^*^R.irregularis^* treatment. The central picture (inside the oval circle) predicts the binding sites of 9 flavonoids in NodD2 protein of *S. meliloti*. Individual figures are shown below. A: sagittain B1 and sagittain B2; B: isosakuranetin-hexosyl-deoxyhexose; C: luteolin-acetylhexuronide; D: taxifolin-deoxyhexosyl-oxanepentol; E: haploside C; F: sudachiin A; G: tetrahydroxy-tetramethoxyflavone-hexoside; H: trihydroxy-flavanone-hexosyl-pentose; I: trimethoxy-hydroxy-methyl-flavanone-dihexosyl-deoxyhexose. Sagittain B1 and Sagittain B2 were merged into a single flavonoid since their comparison results in the PubChem ([https://pubchem.ncbi.nlm.nih.gov](https://pubchem.ncbi.nlm.nih.gov/)) were identical. The figure was designed according to Bag et al. [1].

**Fig. S13** In silico binding studies of NodD2 protein of *S. meliloti* and 10 flavonoids. Each NodD2-flavonoid complex form hydrogen (green dotted line between amino acid and flavonoid, with distances) and hydrophobic (red half circles) bond during NodD2-flavonoid interaction. Sagittain B1 and Sagittain B2 were merged into a single flavonoid since their comparison results in the PubChem (<https://pubchem.ncbi.nlm.nih.gov/>) were identical.

**Fig. S14** In silico binding studies between NodD3 protein of *S. meliloti* and 10 flavonoids. Here NodD3–flavonoid complexes were arranged based on their binding affinities (the more negative the values, (Kcal·mol^−1^), the stronger the interactions). Each NodD3–flavonoid complex form hydrogen (green dotted line between amino acid and flavonoid) and hydrophobic (red half circles) bonds during NodD3–flavonoid interaction. The red-boxed NodD3–flavonoid indicates the flavonoid found exclusively in the RC*^S.tuberosum^*/HC^+^*^R.irregularis^* treatment. The central picture (inside the oval circle) predicts the binding sites of 9 flavonoids in NodD3 protein of *S. meliloti*. Individual figures are shown below. A: sagittain B1 and sagittain B2; B: isosakuranetin-hexosyl-deoxyhexose; C: luteolin-acetylhexuronide; D: taxifolin-deoxyhexosyl-oxanepentol; E: haploside C; F: sudachiin A; G: tetrahydroxy-tetramethoxyflavone-hexoside; H: trihydroxy-flavanone-hexosyl-pentose; I: trimethoxy-hydroxy-methyl-flavanone-dihexosyl-deoxyhexose. Sagittain B1 and Sagittain B2 were merged into a single flavonoid since their comparison results in the PubChem ([https://pubchem.ncbi.nlm.nih.gov](https://pubchem.ncbi.nlm.nih.gov/)) were identical. The figure was designed according to Bag et al. [1].

**Fig. S15** In silico binding studies of NodD3 protein of *S. meliloti* and 10 flavonoids. Each NodD3-flavonoid complex form hydrogen (green dotted line between amino acid and flavonoid, with distances) and hydrophobic (red half circles) bond during NodD3-flavonoid interaction. Sagittain B1 and Sagittain B2 were merged into a single flavonoid since their comparison results in the PubChem ([https://pubchem.ncbi.nlm.nih.gov](https://pubchem.ncbi.nlm.nih.gov/)) were identical.

**Fig. S16** In silico binding studies between NodD1 protein of *S. meliloti* and 4 flavonoids from Compton et al.’s [5] study. Here NodD1–flavonoid complexes were arranged based on their binding affinities (the more negative the values, (Kcal·mol^−1^), the stronger the interactions). Each NodD1–flavonoid complex form hydrogen (green dotted line between amino acid and flavonoid) and hydrophobic (red half circles) bonds during NodD1–flavonoid interaction. The red-boxed NodD1–flavonoid indicates the flavonoid found exclusively in the RC*^S.tuberosum^*/HC^+^*^R.irregularis^* treatment. The central picture (inside the oval circle) predicts the binding sites of 4 flavonoids in NodD1 protein of *S. meliloti*. Individual figures are shown below. a: luteolin-7-glucoside; b: hyperoside; c: luteolin; d: chrysoeriol. The figure was designed according to Bag et al. [1].

**Fig. S17** In silico binding studies of NodD1 protein of *S. meliloti* and 4 flavonoids from Compton et al.’s [5] study. Each NodD1-flavonoid complex form hydrogen (green dotted line between amino acid and flavonoid, with distances) and hydrophobic (red half circles) bond during NodD1-flavonoid interaction.

**Fig. S18** In silico binding studies between NodD2 protein of *S. meliloti* and 4 flavonoids from Compton et al.’s [5] study. Here NodD2–flavonoid complexes were arranged based on their binding affinities (the more negative the values, (Kcal·mol^−1^), the stronger the interactions). Each NodD2–flavonoid complex form hydrogen (green dotted line between amino acid and flavonoid) and hydrophobic (red half circles) bonds during NodD2–flavonoid interaction. The red-boxed NodD2–flavonoid indicates the flavonoid found exclusively in the RC*^S.tuberosum^*/HC^+^*^R.irregularis^* treatment. The central picture (inside the oval circle) predicts the binding sites of 4 flavonoids in NodD2protein of *S. meliloti*. Individual figures are shown below. a: luteolin-7-glucoside; b: hyperoside; c: luteolin; d: chrysoeriol. The figure was designed according to Bag et al. [1].

**Fig. S19** In silico binding studies of NodD2 protein of *S. meliloti* and 4 flavonoids from Compton et al.’s [5] study. Each NodD2-flavonoid complex form hydrogen (green dotted line between amino acid and flavonoid, with distances) and hydrophobic (red half circles) bond during NodD2-flavonoid interaction.

**Fig. S20** In silico binding studies between NodD3 protein of *S. meliloti* and 4 flavonoids from Compton et al.’s [5] study. Here NodD3–flavonoid complexes were arranged based on their binding affinities (the more negative the values, (Kcal·mol^−1^), the stronger the interactions). Each NodD3–flavonoid complex form hydrogen (green dotted line between amino acid and flavonoid) and hydrophobic (red half circles) bonds during NodD3–flavonoid interaction. The red-boxed NodD3–flavonoid indicates the flavonoid found exclusively in the RC*^S.tuberosum^*/HC^+^*^R.irregularis^* treatment. The central picture (inside the oval circle) predicts the binding sites of 4 flavonoids in NodD3 protein of *S. meliloti*. Individual figures are shown below. a: luteolin-7-glucoside; b: hyperoside; c: luteolin; d: chrysoeriol. The figure was designed according to Bag et al. [1].

**Fig. S21** In silico binding studies of NodD3 protein of *S. meliloti* and 4 flavonoids from Compton et al.’s [5] study. Each NodD3-flavonoid complex form hydrogen (green dotted line between amino acid and flavonoid, with distances) and hydrophobic (red half circles) bond during NodD3-flavonoid interaction.

**References**

1. Bag S, Mondal A, Majumder A, Mondal SK, Banik A. Flavonoid mediated selective cross-talk between plants and beneficial soil microbiome. *Phytochem Rev*. 2022; 21:1739–1760.

2. Schrödinger, LLC. The PyMOL molecular graphics system, version 1.8. 2015.

3. Morris GM, Huey R, Lindstrom W, Sanner MF, Belew RK, Goodsell DS, et al. AutoDock4 and AutoDockTools4: automated docking with selective receptor flexibility. *J Comput Chem*. 2009; 30:2785–2791.

4. Laskowski RA, Swindells MB. LigPlot+: multiple ligand–protein interaction diagrams for drug discovery. *J Chem Inf Model*. 2011; 51:2778–2786.

5. Compton KK, Hildreth SB, Helm RF, Scharf BE. An Updated Perspective on S*inorhizobium meliloti* Chemotaxis to Alfalfa Flavonoids. *Front Microbiol*. 2020; 11:581482.
